# Co‐Doping Approach for Enhanced Electron Extraction to TiO_2_ for Stable Inorganic Perovskite Solar Cells

**DOI:** 10.1002/smsc.202400578

**Published:** 2025-05-06

**Authors:** Thomas W. Gries, Davide Regaldo, Hans Köbler, Noor Titan Putri Hartono, Steven P. Harvey, Maxim Simmonds, Chiara Frasca, Marlene Härtel, Gennaro V. Sannino, Roberto Félix, Elif Hüsam, Ahmed Saleh, Regan G. Wilks, Fengshuo Zu, Emilio Gutierrez‐Partida, Zafar Iqbal, Zahra Loghman Nia, Fengjiu Yang, Paola Delli Veneri, Kai Zhu, Martin Stolterfoht, Marcus Bär, Stefan A. Weber, Philip Schulz, Jean‐Baptiste Puel, Jean‐Paul Kleider, Eva Unger, Qiong Wang, Artem Musiienko, Antonio Abate

**Affiliations:** ^1^ Helmholtz‐Zentrum Berlin für Materialien und Energie (HZB) 14109 Berlin Germany; ^2^ Institut Photovoltaïque d’Île‐de‐France (IPVF) 91120 Palaiseau France; ^3^ Laboratoire de Génie Electrique et Electronique de Paris Université Paris‐Saclay CentraleSupélec CNRS 91192 Gif‐sur‐Yvette France; ^4^ Laboratoire de Génie Electrique et Electronique de Paris Sorbonne Université CNRS 75005 Paris France; ^5^ Chemistry and Nanoscience Center National Renewable Energy Laboratory Golden Colorado 80401 USA; ^6^ Portici Research Center Italian National Agency for New Technologies Energy and Sustainable Economic Development (ENEA) 80055 Portici Italy; ^7^ Department of Physics & IRIS Adlershof Humboldt University 12489 Berlin Germany; ^8^ Institute for Physics and Astronomy University of Potsdam 14476 Potsdam Germany; ^9^ Electronic Engineering Department The Chinese University of Hong Kong Sha Tin 999077 Hong Kong; ^10^ Department of Physical Chemistry II Friedrich‐Alexander‐University Erlangen‐Nürnberg 91058 Erlangen Germany; ^11^ Department of X‐Ray Spectroscopy at Interfaces of Thin Films Helmholtz‐Institute Erlangen‐Nürnberg for Renewable Energy (HI ERN) 91058 Erlangen Germany; ^12^ Institute for Photovoltaics University of Stuttgart 70569 Stuttgart Germany; ^13^ École Polytechnique IPVF UMR 9006 CNRS 91120 Palaiseau France; ^14^ Research & Development Électricité de France (EDF) Palaiseau 91120 France; ^15^ Department of Chemistry & IRIS Adlershof Humboldt University 12489 Berlin Germany; ^16^ Faculty of Chemistry University of Bielefeld 33615 Bielefeld Germany; ^17^ Department of Chemical Materials and Production Engineering University of Naples Federico II 80125 Naples Italy

**Keywords:** 2D drift‐diffusion model, CsPbI_3_ solar cells, solar cell stability, surface photovoltage, TiO_2_ co‐doping

## Abstract

Inorganic perovskite CsPbI_3_ solar cells hold great potential for improving the operational stability of perovskite photovoltaics. However, electron extraction is limited by the low conductivity of TiO_2_, representing a bottleneck for achieving stable performance. In this study, a co‐doping strategy for TiO_2_ using Nb(V) and Sn(IV), which reduces the material's work function by 80 meV compared to Nb(V) mono‐doped TiO_2_, is introduced. To gain fundamental understanding of the processes at the interfaces between the perovskite and charge‐selective layer, transient surface photovoltage measurements are applied, revealing the beneficial effect of the energetic and structural modification on electron extraction across the CsPbI_3_/TiO_2_ interface. Using 2D drift‐diffusion simulations, it is found that co‐doping reduces the interface hole recombination velocity by two orders of magnitude, increasing the concentration of extracted electrons by 20%. When integrated into n–i–p solar cells, co‐doped TiO_2_ enhances the projected *T*_S80_ lifetimes under continuous AM1.5G illumination by a factor of 25 compared to mono‐doped TiO_2_. This study provides fundamental insights into interfacial charge extraction and its correlation with operational stability of perovskite solar cells, offering potential applications for other charge‐selective contacts.

## Graphical abstract

TiO_2_ is highly relevant in photoelectrochemistry, (photo)catalysis, and sensor applications, where high conductivity is crucial. Herein, a co‐doping strategy for TiO_2_ using Nb(V) and Sn(IV) is developed, enhancing electron extraction from perovskite and improving solar cell efficiency and stability. Using transient surface photovoltage and drift‐diffusion simulations, buried interfaces are characterized and critical charge transport parameters for optoelectronic advancements are extracted.

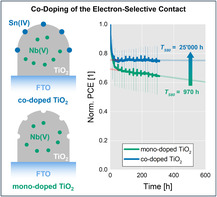

## 1 Introduction

The wide‐bandgap semiconductor TiO_2_ in its anatase crystal phase is generally suitable as an electron‐selective contact (ESC) in perovskite solar cells (PSCs) due to its wide bandgap (*E*_g_) of 3.2 eV and its electron affinity around –4.0 eV against the vacuum level (*E*_vac_).^[^ ^1^ ^]^ However, defects at TiO_2_ surfaces, especially surface oxygen vacancies and Ti(III)‐derived states, serve as non‐radiative recombination centers and are suspected of serving as sites for oxidation of iodide to elemental iodine under irradiation, potentially leading to degradation of the adjacent perovskite.^[^ ^2^ ^]^ Therefore, minimization of surface defects is needed to improve charge extraction and the stability of the resulting PSC. Moreover, the low electron conductivity of pristine TiO_2_ results in increased series resistance and, therefore, reduces the fill factor (FF) of photovoltaic devices.^[^ ^3^ ^]^

Stable operation is one of the critical factors for the successful market introduction of PSCs. To achieve stable operation, photoinduced degradation processes in the perovskite light‐absorbing layer must be minimized. Organic cations, such as methylammonium (MA^+^), are prone to intrinsic destabilization of the perovskite absorber due to their volatile nature.^[^ ^4^ ^]^ From this perspective, fully inorganic perovskites, such as CsPbI_3_ (*E*_g_ = 1.7 eV), are attractive candidates for achieving stable PSCs.[^4^, ^5^] Best‐performing n–i–p‐structured PSCs based on inorganic CsPbI_3_ currently use TiO_2_ or SnO_2_ as ESC and 2,2′,7,7′‐tetrakis(*N*,*N*‐di‐4‐methoxyphenylamino)‐9,9′‐spirobifluorene (spiro‐OMeTAD) as hole‐selective contact (HSC).^[^ ^6^ ^]^

In our study, we introduce a strategy for co‐doping TiO_2_, reducing the defect density at the ESC/CsPbI_3_ interface. Aliovalent doping of anatase TiO_2_ with Nb(V) is an established procedure for enhanced n‐type characteristics of the material.^[^ ^7^ ^]^ Nb(V) enhances the material's conductivity by releasing additional electrons into the conduction band.[^7^, ^8^] Moreover, Nb(V) inhibits the transition to undesirable rutile TiO_2_.^[^ ^9^ ^]^ Nb(V)‐doped TiO_2_ was first applied in dye‐sensitized solar cells in 2010 and in PSCs in 2015, where the improved photovoltaic performance at a doping level of 0.5% Nb(V) was ascribed to suppressed Ti(III) defect creation.^[^ ^10^ ^]^ However, the achievable upward shift of the Fermi level (*E*_F_) in anatase TiO_2_ by using Nb(V) is limited since *E*_F_ decreases again beyond the maximum value, possibly due to localized Ti 3d^1^ states in highly Nb(V)‐doped TiO_2_.[^10^, ^11^] In this study, we add isovalent Sn(IV) as a co‐dopant to Nb(V)‐doped TiO_2_ to further decrease the work function (WF) of TiO_2_. By combining both dopants, we surpass the limit of the Nb(V) mono‐dopant.

Characterization of buried interfaces is crucial for understanding charge carrier dynamics.^[^ ^12^ ^]^ To unveil the effect of co‐doping on the fundamental charge extraction and recombination mechanisms, we applied transient surface photovoltage (trSPV) and drift‐diffusion (DD) modeling. Recorded SPV transients are frequently assessed qualitatively.^[^ ^12^, ^13^ ^]^ However, quantitative elucidation of extraction and recombination velocities requires precise knowledge of the material energetics and advanced curve fitting models. So far, the minimalistic kinetic model developed by Levine and Musiienko is often used.[^6^, ^12^, ^14^] The minimalistic kinetic model, however, lacks incorporation of drift and diffusion phenomena, consideration of the alignment of interfaces, and the spatial distribution of charge carriers. These drawbacks restrict our ability to acquire comprehensive insights into interface characteristics through time‐resolved measurements. Here, we developed an approach to fitting SPV transients with the DD model, including drift and diffusion phenomena. With the help of the DD model, we reveal the fundamental properties of electron extraction and recombination. We show that TiO_2_ co‐doped with 0.5% Nb(V) and 0.1% Sn(IV) exhibits strongly reduced recombination of electrons with hole minority carriers. This reduction is expressed in an interfacial hole recombination velocity, *ν*_interf,h_, of 0.098 cm s^−1^, representing a reduction by two orders of magnitude compared to 17.0 cm s^−1^ for TiO_2_ mono‐doped with 0.5% Nb(V). Consequently, the concentration of extracted electrons is enhanced from 3.7 to 4.8 × 10^10^ cm^−2^, respectively, as detailed later.

An improved PSC performance reflects the lower interfacial hole recombination velocity and increased number of extracted electrons for co‐doped TiO_2_. The power conversion efficiency (PCE) is enhanced by 1.0% absolute on average, mainly originating from a statistically relevant improvement in FF by 2% to 80.8%. Further, the open‐circuit voltage (*V*_OC_) is slightly improved by 10 mV to 1.18 V. The lowered extraction barrier in co‐doped TiO_2_ and the reduced hole recombination velocities are responsible for both FF and *V*_OC_ improvement.^[^ ^15^ ^]^ Most importantly, the co‐doping approach stabilizes maximum power point (MPP) tracking under continuous AM1.5G illumination, where no degradation is detected within the first 300 h. As a result, the linearly projected *T*_S80_ lifetime improves from 970 h in mono‐doped TiO_2_ to 25,000 h in co‐doped TiO_2_.

## 2 Results and Discussion

### 2.1 Energetic and Structural Aspects of Co‐Doping the ESC

Doped TiO_2_ ESCs were deposited using the spray pyrolysis technique. The process yielded compact layers of 20 nm thickness, as confirmed by variable‐angle spectroscopic ellipsometry (VASE, Figure S1, Supporting Information). To quantify the energetic effect of dopant addition on the WF of the as‐deposited TiO_2_, we measured the surface potential via Kelvin‐probe force microscopy (KPFM) in the dark. The details about the measurement conditions and data treatment are given in Section S3.2, Supporting Information. The choice of dopant concentrations was based on photovoltaic performance, as shown in Figure S17 and S18, Supporting Information.

**Figure 1:**
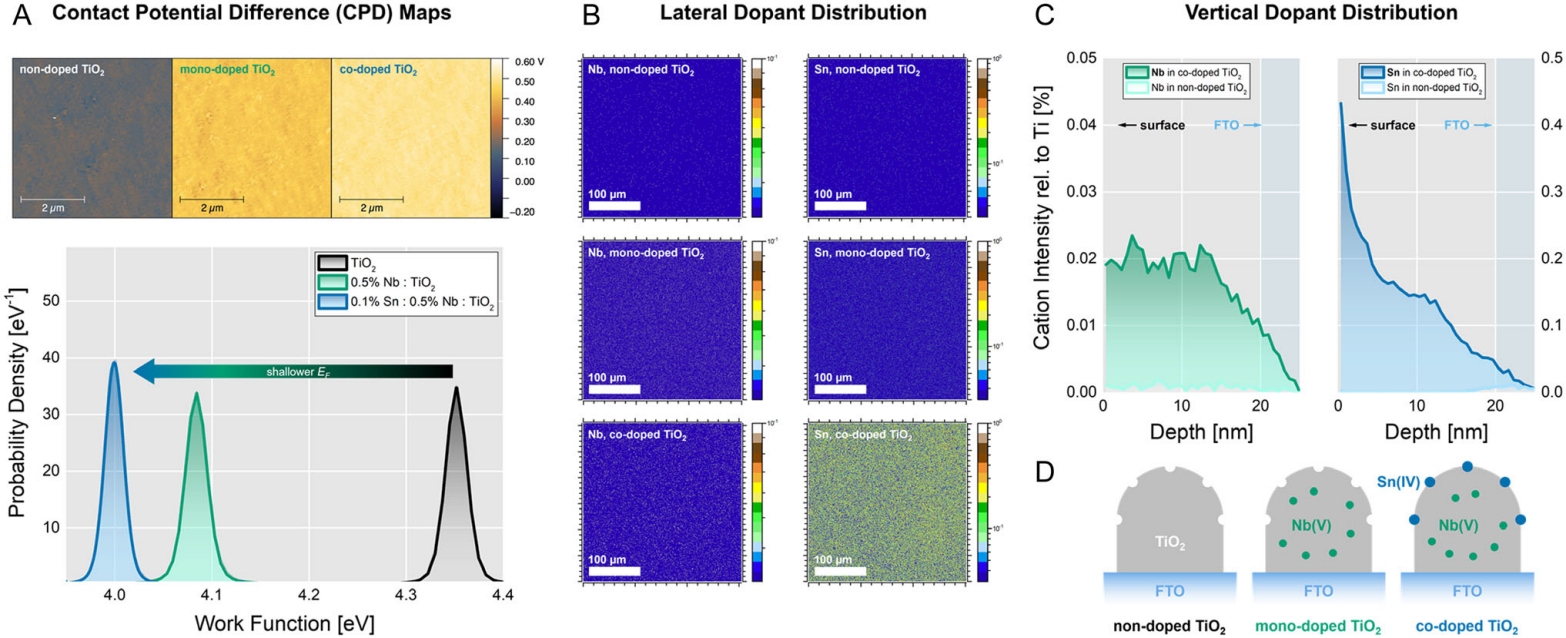
The WF of TiO_2_ can be tuned via co‐doping with Nb(V) and Sn(IV). A) The maximum reduction of the WF of Nb(V) mono‐doped TiO_2_ can be overcome by co‐doping with Sn(IV). The CPD distributions are extracted from (5 × 5) μm^2^ KPFM scans in argon atmosphere, minimizing the influence of water adsorption on the measured WF values. B) Homogeneous lateral dopant distribution was confirmed via ToF‐SIMS imaging of (doped) TiO_2_ layers fabricated on FTO. C) ToF‐SIMS depth profiles reveal a homogeneous vertical distribution of the Nb(V) dopant in the crystal structure of TiO_2_ (left), while the Sn(IV) dopant is predominantly located at the TiO_2_ surface (right). D) Schematic of non‐doped TiO_2_ (left), mono‐doped TiO_2_ with homogeneously distributed Nb(V) (middle), and co‐doped TiO_2_ with additional surficial Sn(IV) (right).

With Nb(V)‐doping alone, a reduction of the WF by 270 meV could be achieved at a concentration of 0.5% Nb(V), as shown in **Figure** **1**A. The same dopant concentration was confirmed as an optimum in other studies, although changes in WF have not been quantified.^[^ ^10^ ^]^ Further, after adding 0.1% of co‐dopant Sn(IV), we observed an even lower WF by 80 meV compared to the mono‐doped champion. Assuming vacuum level alignment, the reduction of the WF can be interpreted as a rise in *E*_F_. In both cases, the WF increases again beyond the optimum dopant concentrations (Figure S4, Supporting Information). This increase may be associated with the creation of intragap defect states, such as the Ti 3d^1^ state for Nb(V)‐doped anatase mentioned earlier or, in the case of Sn(IV) doping, a facilitated phase transition to rutile TiO_2_, which exhibits a lower *E*_F_.^[^ ^16^ ^]^ We will refer to pristine TiO_2_ as *non‐doped* TiO_2_, to TiO_2_ doped with 0.5% Nb(V) as *mono‐doped* TiO_2_, and to TiO_2_ doped with 0.1% Sn(IV) and 0.5% Nb(V) as *co‐doped* TiO_2_ hereinafter. Beyond quantification of the average shift of the WF, effects such as phase segregation and transformation are indicated in the full‐width at half‐maximum of the contact potential difference (CPD) histograms, which were obtained on (5 × 5) μm^2^ KPFM scans (Figure 1A). Compared to the reference line width of 25 meV obtained on non‐doped TiO_2_, only the sample over‐doped with 2.0% Sn(IV) showed significant broadening (Figure S4, Supporting Information). As suggested earlier, this broadening may be caused by the phase transition to rutile TiO_2_ promoted by Sn(IV). In all other samples, the absence of line broadening indicates a homogenous phase formation without segregation phenomena.

The WF of 3.49 eV for CsPbI_3_ measured with KPFM in the dark is comparable to values measured with ultraviolet photoelectron spectroscopy reported elsewhere (for details, see Section S3.2, Supporting Information).^[6d]^ By co‐doping TiO_2_, the energetic misalignment to CsPbI_3_ is expected to be reduced.^[^ ^17^ ^]^ In addition, the shift of *E*_F_ toward the conduction band minimum level (*E*_CBM_) may indicate an enhanced conductivity of TiO_2_.

To investigate the structural origin of the reduction of the WF in mono‐ and co‐doped TiO_2_, we measured time‐of‐flight secondary‐ion mass spectrometry (ToF‐SIMS). Nb(V)‐related signal is absent in non‐doped TiO_2_, while mono‐ and co‐doped TiO_2_ exhibit homogenous lateral Nb(V) distribution across the tested (50 × 50) μm^2^ surface areas, as shown in Figure 1B. A homogeneous dopant distribution is consistent with the narrow width of the CPD distributions in KPFM. The recorded ToF‐SIMS depth profiles (Figure 1C) additionally show homogeneous vertical Nb(V) distribution, indicating full integration of Nb(V) into the TiO_2_ crystal structure, which is consistent with the results of previous studies.^[7a]^ To resolve the vertical distribution of Sn(IV) in co‐doped TiO_2_, we deconvoluted the Sn‐related signal contribution of the dopant from the contribution of the substrate, as described in detail in Section S3.3, Supporting Information. In contrast to Nb(V), the deconvolution of Sn‐related signal reveals a predominant localization of the Sn(IV) dopant atoms on the surface of TiO_2_ (Figure 1C). We therefore suggest that Sn(IV) is not fully integrated into the TiO_2_ crystal structure due to its larger ionic radius of 69.0 pm compared to 64.0 pm for Nb(V) and 60.5 pm for Ti(IV).^[^ ^18^ ^]^ Instead, Sn(IV) may occupy metal vacancies on the TiO_2_ surface, effectively contributing to a reduction of the surface trap‐state density of TiO_2_.

The chemical structure at the surface of the (co‐)doped sample series was further investigated via synchrotron‐based hard X‐ray photoelectron spectroscopy (HAXPES), allowing for a more significant information depth than laboratory‐based X‐ray photoelectron spectroscopy (XPS) instruments. This allowed us to study the dopant content and its impact on the bulk of the samples in the Ti chemical environment (near‐surface). For this purpose, thin films of TiO_2_ (i.e., 20 nm nominal thickness) with corresponding (co‐)doping levels were deposited on fluorine‐doped tin oxide (FTO)‐coated glass substrates (further sample preparation details are described in Section S2.3, Supporting Information). The HAXPES survey spectra of the investigated samples are shown in Figure S10, Supporting Information. Inspecting the survey spectra reveals that the signal detected from the samples originates predominantly from the TiO_2_ layers; however, a minor (yet significant) and varying fraction of the signal derives from the FTO substrate, indicating an incomplete coverage. As the FTO contains Sn(IV), we refrain from assessing the chemical environment and quantity of the Sn(IV) dopant, especially considering the low Sn(IV) (co‐)doping levels (i.e., 0.1% and 2%) used in this study. Figure S11, Supporting Information, shows HAXPES detail spectra of the A) Ti 2p and B) Nb 3d energy regions of the investigated samples series, measured with 2 keV excitation, including curve fit results (for further details on the curve fit analysis of measured spectra, see Section S1, Supporting Information). As shown in Figure S11A, Supporting Information, the binding energy (BE) position of the Ti 2p_3/2_ line is found at (459.3 ± 0.1) eV, consistent with reports for TiO_2_ in literature.^[^ ^19^ ^]^ Moreover, no discernible signal is detected at BE ranges reported for Ti(III) (e.g., Ti_2_O_3_) or Ti(II) (e.g., TiO) in literature,^[^ ^19^, ^20^ ^]^ as denoted by the gray‐filled areas in the figure. The highly similar line shape of the Ti 2p spectra for all investigated samples, with only one set of doublet peaks needed to obtain good fit results, indicates a homogenous Ti chemical environment throughout the sample series, comprising one Ti chemical species (i.e., TiO_2_). Likewise, the Nb 3d spectra of all Nb‐doped samples, as shown in Figure S11B, Supporting Information, can be modeled with one set of doublet peaks, indicating one shared Nb chemical environment. The Nb 3d_5/2_ line is located at a BE value of (207.8 ± 0.1) eV, matching reports of Nb(V) (e.g., Nb_2_O_5_) in literature.^[^ ^19^ ^]^ Based on the intensities of the Ti 2p and Nb 3d measurements, the [Nb]:[Ti] composition ratio of the samples can be computed (for further details on HAXPES‐derived quantification, see Section S1, Supporting Information). The results of this quantification are shown in Figure S11C, Supporting Information, which are in excellent agreement with the nominal Nb(V)‐doping concentration.

The KPFM maps show lateral WF homogeneity, suggesting that the derived WF values are not influenced by the coverage issues observed using HAXPES. Additionally, lateral and vertical homogeneity of Nb(V) in TiO_2_ was confirmed via ToF‐SIMS. ToF‐SIMS further revealed predominant localization of Sn(IV) at the surface of co‐doped TiO_2_, justifying the decreased WF of TiO_2_ upon addition of 0.5% Nb(V) and 0.1% Sn(IV), and potentially reducing the surface trap‐state density. HAXPES Ti 2p results show no discernible Ti(III) contribution for these samples (Figure S11A, Supporting Information). Therefore, doping does not affect the density of Ti(III) defect states. The associated increase in conductivity reduces the series resistance and mainly leads to an increase in the FF of respective solar cells, as discussed later. Next, we focus on the interfacial charge carrier dynamics of the doped TiO_2_ systems contacted in a heterojunction with CsPbI_3_ perovskite.

### 2.2 Interface Characterization

To fully characterize the impact of (co‐)doping on non‐radiative recombination and charge extraction dynamics, we measured absolute steady‐state photoluminescence (ssPL), transient photoluminescence (trPL), and trSPV on heterojunctions of (doped) TiO_2_ and CsPbI_3_. The perovskite preparation and crystallization were equal in all cases, as evidenced by an unchanged film morphology confirmed via scanning electron microscopy (SEM, Figure S12, Supporting Information) and an unchanged film crystallinity confirmed via X‐ray diffraction (XRD, Figure S13, Supporting Information).

**Figure 2:**
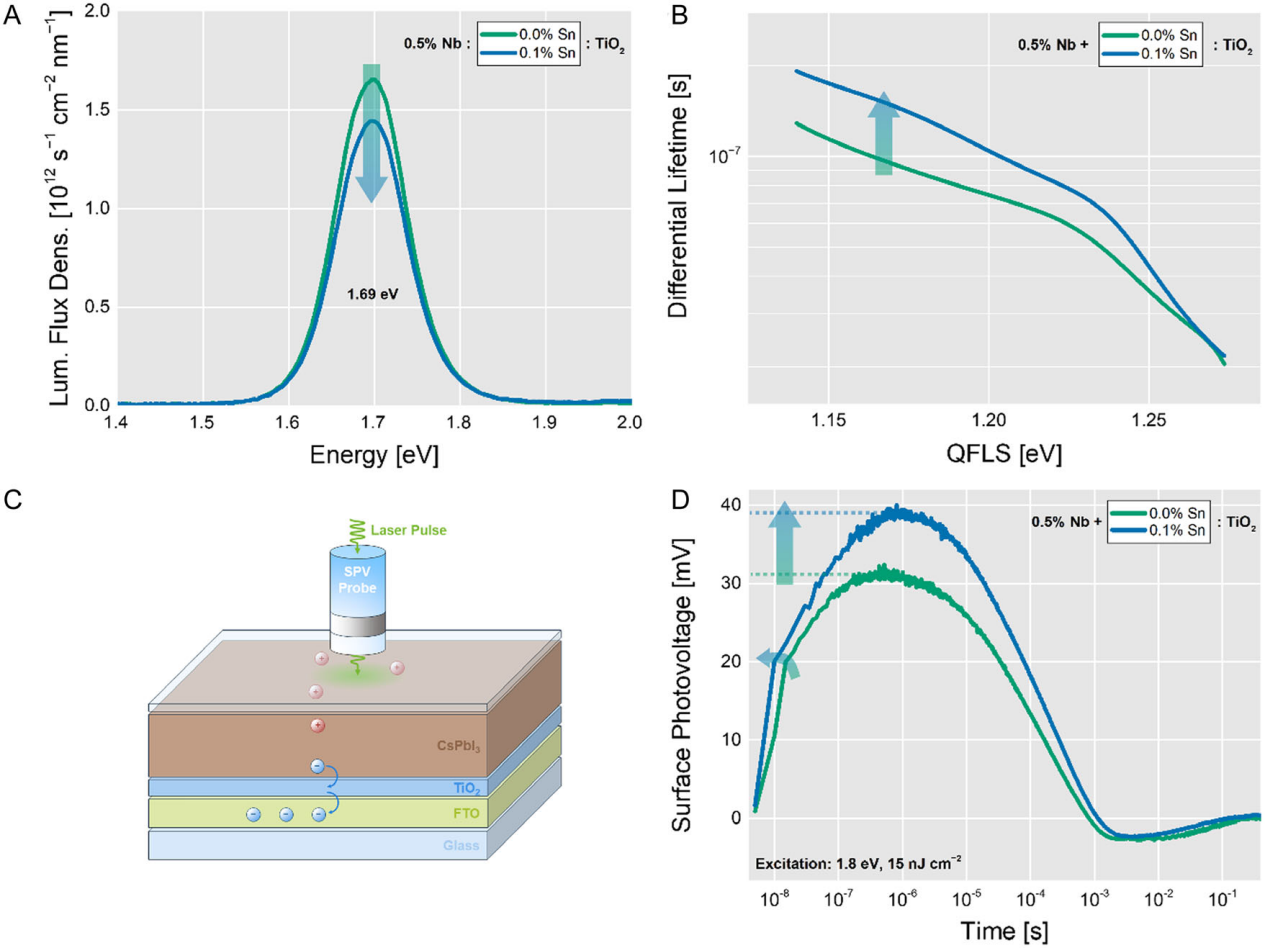
Co‐doping of TiO_2_ with Nb(V) and Sn(IV) reduces non‐radiative recombination and improves the charge extraction efficiency. A) ssPL intensity decreases upon co‐doping 0.5% Nb(V) TiO_2_ with 0.1% Sn(IV). B) Differential lifetime analysis of trPL curves reveal charge carrier lifetimes of 21 ns for CsPbI_3_ on mono‐ and co‐doped TiO_2_ after excitation. The differential lifetime after initial charge carrier depletion is consistently higher for CsPbI_3_ on co‐doped TiO_2_, implying reduced non‐radiative recombination. C) Illustration of the origin of the SPV signal. Upon excitation, electrons are extracted from CsPbI_3_ to (co‐doped) TiO_2_. The separated charge is detected as a positive signal by the SPV probe in a fixed capacitor arrangement. D) The corresponding trSPV curve shows that co‐doped TiO_2_ extracts more electrons at a higher rate compared to mono‐doped TiO_2_.

We investigated CsPbI_3_ deposited on mono‐doped TiO_2_ and on a concentration series of co‐doped TiO_2_ via ssPL, capturing the reversal of the WF of the ESC beyond 0.1% Sn(IV) observed in KPFM. Increasing the co‐dopant level of Sn(IV) leads to a decrease in the photoluminescence (PL) intensity (**Figure** **2**A and S13C, Supporting Information), synonymous with decreased radiative recombination. However, the decrease in radiative recombination may not only originate from an increase of non‐radiative recombination due to increased interface trap‐state density but also due to the conductivity of the charge‐selective contact and associated changes in charge extraction from CsPbI_3_.^[^ ^21^ ^]^ Given that the lowest WF was found for TiO_2_ co‐doped at 0.1% Sn(IV), we suggest that interface non‐radiative recombination increases with respect to mono‐doped TiO_2_ due to the higher conductivity of the ESC. In contrast, in the mono‐doped case and at higher co‐doping levels, the increase in non‐radiative recombination is driven by a higher interface trap‐state density, which is also supported by the broadening of the WF distribution for TiO_2_ over‐doped at 2.0% Sn(IV) (Figure S4, Supporting Information). Since ssPL measurements alone cannot provide detailed insights into the interfacial charge‐carrier dynamics, we employed time‐resolved spectroscopic methods for further characterization.

We analyzed the transient decay of the PL signal obtained in trPL (Figure S15A, Supporting Information) via differential lifetime analysis (Figure 2B), which quantifies the charge carrier lifetime based on quasi‐Fermi level splitting (QFLS), as described in detail in Section S3.8, Supporting Information. Immediately after excitation, QFLS is the highest and subsequently decreases during recombination. At high QFLS, CsPbI_3_ on co‐doped TiO_2_ exhibits a differential lifetime of 21 ns, compared to CsPbI_3_ on mono‐doped TiO_2_ (Figure 2B). The initial depletion of charge carriers is influenced both by electron extraction and fast non‐radiative recombination, both of which are difficult to disentangle.^[^ ^22^ ^]^ However, as QFLS depletes, the differential lifetime of CsPbI_3_ on co‐doped TiO_2_ remains consistently higher than for CsPbI_3_ on mono‐doped TiO_2_. Since changes in differential lifetime resulting from changes in crystal quality are not to be expected, the improvement in differential lifetime is attributed to a lower interfacial trap density in the heterojunction of co‐doped TiO_2_ and CsPbI_3_.

Due to the difficulty of disentangling the contributions of fast non‐radiative recombination and electron extraction to the initial differential lifetime at high QFLS, we employed trSPV measurements to directly measure charge‐separating phenomena at the TiO_2_/CsPbI_3_ interface. In trSPV experiments on TiO_2_/CsPbI_3_ heterojunctions (Figure 2C), the signal amplitude is proportional to the charge extracted (Figure 2D). The time resolution allows for a differentiation of extracted charge carriers from other phenomena creating separated charges, such as (de‐)trapping, recombination, or ionic displacement.^[^ ^23^ ^]^ We show trSPV curves of mono‐doped and co‐doped TiO_2_/CsPbI_3_ samples in Figure 2D. The initial slope in the nanosecond‐regime of the transient curve is proportional to the extraction rate across the TiO_2_/CsPbI_3_ interface. Additionally, the absolute amplitude is directly correlated to the maximum charge separation possible within the heterojunction at the charge carrier density induced by the laser pulse (laser fluence *F*_laser_ = 15.0 nJ cm^−2^). Co‐doped TiO_2_/CsPbI_3_ exhibits the highest extraction rate as well as the largest charge separation. Although CsPbI_3_ on mono‐ and co‐doped TiO_2_ exhibited similar initial differential lifetimes in trPL, trSPV confirms a higher contribution of charge extraction to the fast processes in co‐doped TiO_2_ while, in mono‐doped TiO_2_, non‐radiative recombination contributes to the reduction of differential lifetime to a higher degree.

Interestingly, the trSPV curves of doped TiO_2_ in Figure 2D exhibit an overshoot below zero at millisecond timescales. We hypothesize that the signal can be assigned to the capture of electrons in deep trap states. Due to their depth, de‐trapping times are significantly longer than for shallow trap states. After the full back‐transfer of electrons from the ESC and recombination, the remaining captured electrons are responsible for the SPV signal in the opposite direction. The overshoot of the signal is not influenced by our co‐doping strategy, implying that those deep trap states are located on the exposed surface of the perovskite, potentially being the reason for hysteresis frequently observed in full devices based on CsPbI_3_.[^6^, ^17^, ^24^]

We demonstrated via ToF‐SIMS that Sn(IV) is predominantly localized at the very surface of co‐doped TiO_2_. The Sn(IV) surface localization may reduce the surface trap‐state density and is likely the reason for the reduced WF compared to mono‐doped TiO_2_, as observed in KPFM. Differential lifetime analysis of trPL data revealed generally longer charge carrier lifetimes in CsPbI_3_ on co‐doped TiO_2_, which we associated with a reduced non‐radiative recombination rate due to a lower interface trap‐state density, originating from the co‐doped TiO_2_ structure as elucidated by ToF‐SIMS.

Energetically, a changed WF may have several consequences on the TiO_2_ properties, such as a change of occupation or concentration of defects,^[^ ^25^ ^]^ or the concentration of free electrons in TiO_2_. Both factors influence the non‐radiative recombination rate. While the decrease of the WF and associated increase in conductivity in co‐doped TiO_2_ may cause faster non‐radiative recombination rates, this acceleration is mitigated by a lower interface trap‐state density. Inversely, a higher WF and a higher interface trap‐state density for mono‐doped TiO_2_ may lead to initial charge carrier lifetimes in trPL that are similar to those of co‐doped TiO_2_. We confirmed via trSPV that electron extraction from CsPbI_3_ to co‐doped TiO_2_ is improved compared to mono‐doped TiO_2_, likely due to the reduced interface trap‐state density. Since trSPV measurements primarily provide qualitative rather than quantitative insights, we developed a simulation approach based on DD to extract crucial parameters from the recorded trSPV curves.

### 2.3 Simulation of TrSPV Curves

By using KPFM, ToF‐SIMS, PL, and SPV, we demonstrated that the charge extraction across the TiO_2_/CsPbI_3_ interface is improved via co‐doping of TiO_2_ with Nb(V) and Sn(IV), possibly due to an increase in conductivity and a reduced interface trap‐state density. To fully interpret the trSPV data in terms of carrier photogeneration, diffusion, and extraction, we employed 2D DD simulations. We report more details on the simulations in Supporting Information, together with the applied parameters (Table S5–S8, Supporting Information).

In the DD simulation, we account for the development of the electric field according to Poisson's equation, as described by Equation S10–S15, Supporting Information. Both the trSPV curves of mono‐doped and co‐doped TiO_2_ were fitted with the DD model, which features a non‐doped perovskite layer, a non‐doped but defective TiO_2_ layer, an interface layer between the two, and a Schottky‐type contact at the back of TiO_2_. While certain parameters were common to the fits in the mono‐ and co‐doped cases, such as the perovskite carrier mobility (*μ*_e,h_) and the non‐radiative characteristic time in CsPbI_3_ (*τ*_e,h_). Other parameters, such as the non‐radiative recombination velocity at the TiO_2_/CsPbI_3_ interface (*ν*_interf,e_ and *ν*_interf,h_), and the donor trap parameters in TiO_2_, were fitted independently on each curve.

For both configurations, the band alignment between CsPbI_3_ and TiO_2_ is governed by the electron affinity of the perovskite layer, *χ*_pero_, while the bulk diffusion length depends on mobility and lifetime. In addition to recombination in the bulk, we expect non‐radiative recombination at the TiO_2_/CsPbI_3_ interface, which depends on the TiO_2_ composition since previous works have pointed out the presence of defects on the TiO_2_ surface such as oxygen vacancies and extrinsic adsorbed species.^[^ ^16^, ^26^, ^27^, ^28^, ^29^ ^]^

To model the complex doping picture of TiO_2_, we chose to add single‐energy level (*E*_tD_) bulk donor traps with defined density (*N*_tD_) and electron capture cross section (*σ*_etD_) letting these parameters vary to fit the trSPV curves. These traps modify the carrier concentration in TiO_2_, inducing an n‐type doping nature as well as storing photoelectrons injected into TiO_2_ from CsPbI_3_.

**Figure 3:**
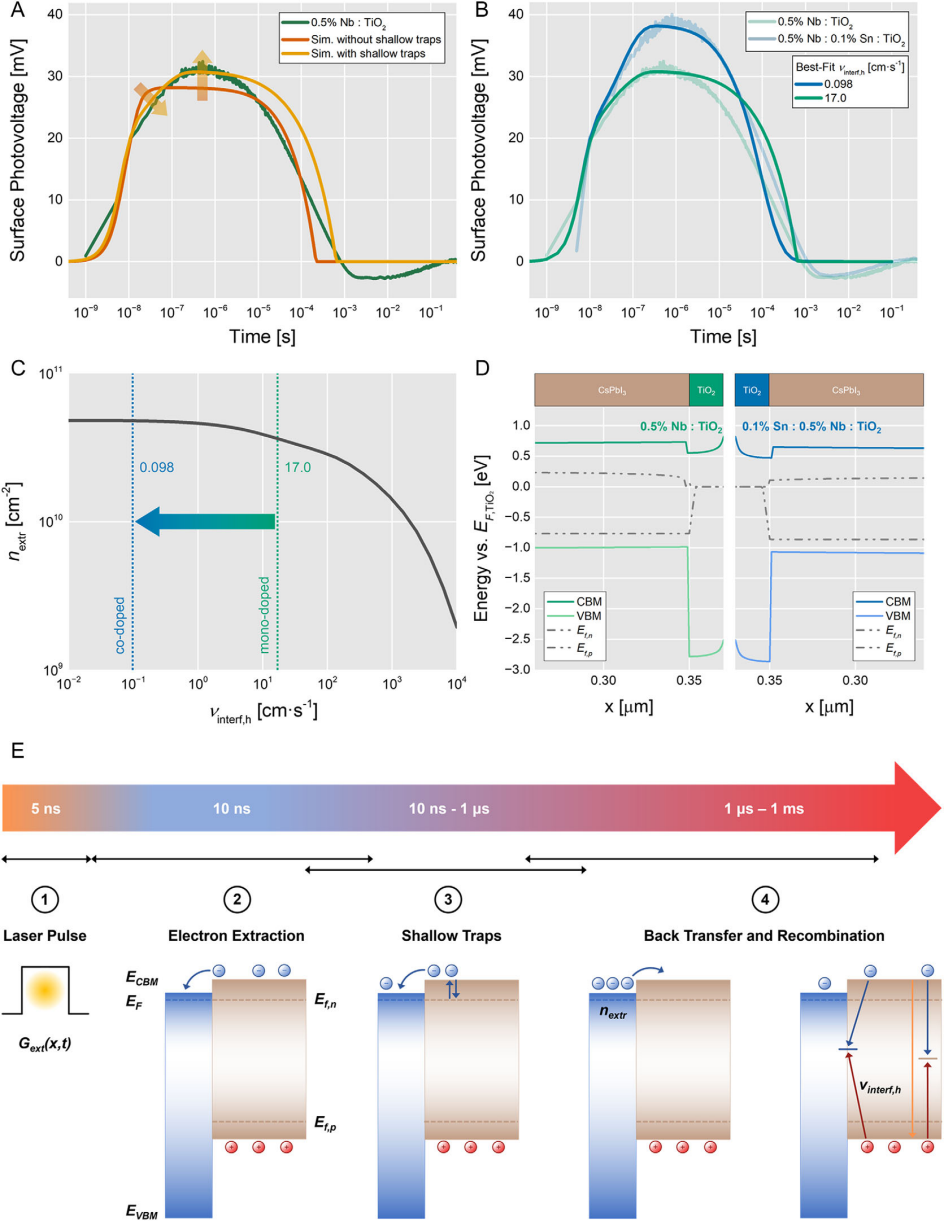
2D DD simulations of recorded trSPV data reveal a reduction of the interface hole recombination velocity by two orders of magnitude. A) The match between the original data (green) and the simulated curve (red) improves after consideration of shallow electron traps in the CsPbI_3_ layer (orange). B) In the simulation model, the signal height is sensitive to the interface hole recombination velocity *ν*_interf,h_. Fitting the original data yields 17.0 cm s^−1^ for mono‐doped TiO_2_ and 0.098 cm s^−1^ for co‐doped TiO_2_. C) Reduction of *ν*_interf,h_ leads to a higher concentration of extracted electrons. D) Calculated out‐of‐equilibrium band diagram at maximum charge separation (SPV peak at *t* = 0.35 μs). E) DD simulations enabled us to associate decelerated electron extraction (10 ns–1 μs) with the presence of shallow traps in CsPbI_3_. For simplicity, the schematic disregards band‐bending effects. 1) At *t* = 0, electrons (blue spheres) and holes (red spheres) are photo‐generated in CsPbI_3_ (brown box). 2) Electron extraction to the TiO_2_ ESC (blue box) is the reason for the charge separation at around 10 ns as measured with trSPV. 3) The kink in the trSPV curve between 10 ns–1 μs implies ongoing charge separation by extraction at decelerated velocity until a maximum of extracted electrons *n*_extr_ is reached. The kink was reproduced by DD simulation via introduction of shallow traps in CsPbI_3_. 4) Signal depletion between 1 μs–1 ms is caused by charge carrier recombination via different pathways, comprising electron back‐transfer and subsequent classical radiative or non‐radiative pathways.

In **Figure** **3**A, we report the fit result on the mono‐doped TiO_2_ trSPV curve, employing the parameters listed in Table S5, Supporting Information. On the experimental curve, we can identify four different regions: a rapid rise of SPV signal at *t* < 10^−8^ s, followed by a slower rise between 10^−8^ s < *t *< 10^−6^ s, a decay of SPV signal between 10^−6^ s < *t* < 10^−3^ s, and a negative tail after *t* > 10^−3^ s. The positive polarity results from an accumulation of positive charges close to the surface, while negative charges are driven toward the bulk. This is in line with the result of our simulations, where the positive sign is due to electron diffusion toward the TiO_2_/CsPbI_3_ interface and subsequent injection into the TiO_2_ layer. At the same time, holes only diffuse toward the buried interface and cannot be injected into the TiO_2_ layer due to the energetic offset. Being governed by diffusion, we can estimate the transit time of electrons across the CsPbI_3_ layer to 33 ns via *τ*_diff_^−1^, where *L*_pero_ is the CsPbI_3_ thickness and *D* is the diffusion coefficient (*D *= *k* *T* *μ*_e,h_ *q* ^−1^, with *k* *T* being the electron thermal energy and *μ*_e,h_ and *q* being the electron mobility and charge, respectively). *τ*_diff_ corresponds to the simulated SPV rise time in Figure 3A. Electron injection in TiO_2_ is allowed by the conduction band alignment between CsPbI_3_ and TiO_2_ and is further favored by the large concentration of deep donor traps in the TiO_2_ layer. These traps create states within the TiO_2_ bandgap that capture and store electrons quickly, allowing further injection of electrons from CsPbI_3_.

While the initial fast rise and decay of the SPV are decently reproduced by our model, the current set of parameters does not yet describe the slow rise after *t* = 10^−8^ s. To improve the fit, we introduced a delay component in the SPV rise, which allowed the SPV to continue growing slowly after the fast rise. This was accomplished by adding shallow electron acceptor traps close to the CBM of CsPbI_3_ (see orange curve in Figure 3A, best‐fit parameters in Table S7, Supporting Information). With the newly found CsPbI_3_ carrier mobility, we find the electron diffusion time across the CsPbI_3_ layer, which coincides with the kink in the experimental trSPV curve at *τ*_diff_ = 12 ns. Simultaneously, shallow traps in CsPbI_3_ capture electrons with an average capture time of (*σ*_etA_ *ν*_th_ *N*_tA_)^−1^ = 10 ns, where *N*_tA_ is the acceptor trap density in CsPbI_3_, *σ*_etA_ is the electron capture cross section associated with the traps, and *ν*_th_ is the electron thermal velocity.

Between 10^−8^ s < *t* < 10^−6^ s, the signal rise is no longer dominated by electron diffusion, but rather by electron de‐trapping from the shallow traps to the conduction band and subsequent diffusion toward TiO_2_. The electron reemission time can be estimated at *τ*_em,pero_ = *σ*_etA_ *ν*_th_ *N*_C_ exp^−1^[(*E*_tA_ − *E*_CBM,pero_) (*k T*)^−1^] = 36 ns, which is close to the SPV peak time. Here, *N*_C_ is the conduction band effective density of states, *E*_tA_ is the bulk acceptor trap level, and (*E*_tA_ − *E*_CBM,pero_) is referring the trap energy to the CBM.

With the same principle, we can estimate the electron capture time *τ*_cap,TiO2_ of TiO_2_ traps by employing the trap density *N*_tD_ and the electron capture cross section *σ*_etD_. We find *τ*_cap_,_TiO2_ = (*σ*_etD_ *ν*_th_ *N*_tD_)^−1^ = 2.7 × 10^−15^ s, implying that it does not limit electron diffusion toward TiO_2_. In addition, we find *τ*_em,TiO2_ = 2.3 × 10^−3^ s for the electron release time from TiO_2_ traps. *τ*_em,TiO2_ roughly corresponds to the SPV decay time in our model since the SPV decay is governed by electron reemission from TiO_2_ traps, followed by recombination across the interface, with holes present in the CsPbI_3_ layer. Having obtained a satisfying fit, we can now evaluate the impact of each parameter on the simulated curve.

We found that the SPV signal height depends mostly on generation and recombination‐related parameters, which control the number of electrons that can be injected into TiO_2_. In particular, it depends on the laser fluence *F*_laser_, on the bulk non‐radiative characteristic time in CsPbI_3_ (*τ*_e,h_) and carrier mobility in CsPbI_3_ (*μ*_e,h_), which control the bulk diffusion length, and *ν*_interf,e_ and *ν*_interf,h_. The signal height also depends on the shallow electron acceptor trap parameters since these defects can recombine electrons with holes in bulk CsPbI_3_ and also store electrons.

Crucially, *ν*_interf,e_ and *ν*_interf,h_ should strongly depend on the TiO_2_ composition. While in our model, *ν*_interf,e_ controls both the SPV amplitude and decay time, *ν*_interf,h_ influences primarily the SPV amplitude. If we compare the trSPV curves of mono‐ versus co‐doped TiO_2_, *ν*_interf,h_ decreases from 17.0 to 0.098 cm s^−1^, with the other parameters set according to Table S7, Supporting Information. In addition, by increasing the TiO_2_ donor trap energy level from 2.673 to 2.758 eV from the TiO_2_ valence band maximum, the fit further improves. The obtained parameter values closely match the best‐fit parameters for co‐doped TiO_2_ found in Table S8, Supporting Information. The best‐fit curves for both mono‐ and co‐doped TiO_2_ are shown in Figure 3B. Therefore, according to our model, the signal height difference between the two curves is mainly due to an improvement of the TiO_2_/CsPbI_3_ interface, which translates into a reduced *ν*_interf,h_ of two orders of magnitude in co‐doped TiO_2_ compared to mono‐doped TiO_2_. Figure S23D, Supporting Information, reports the signal height dependence on *ν*_interf,h_.

The other crucial difference between the mono‐ and co‐doped parameters is the TiO_2_ donor trap energy level. Since these traps control the TiO_2_ equilibrium *E*_F_, a WF difference of 82 meV is found (*ϕ*_mono_ − *ϕ*_co_ = 82 meV), implying that the *E*_F_ of co‐doped TiO_2_ moves closer to the CBM compared to mono‐doped TiO_2_, synonymous with larger n‐type character. This result perfectly agrees with the WF difference experimentally observed in KPFM.

To further consolidate the improvement in the extraction properties, Figure 3C shows the total extracted electron concentration for the two fitted configurations. The reduced *ν*_interf,h_ of 17.0 to 0.098 cm s^−1^ upon Sn(IV) addition translates to extracted electron concentrations of 3.7 to 4.8 × 10^10^ cm^−2^, respectively. In Figure 3D, we report the simulated out‐of‐equilibrium band diagram for *t* = 0.35 μs, representing the SPV peak, for mono‐ and co‐doped TiO_2_. The lack of doping in the CsPbI_3_ layer manifests in the absence of an electric field across the layer, where the equilibrium *E*_F_ is controlled by TiO_2_. If an HSC were deposited on CsPbI_3_, such as in a working PSC, a constant electric field would appear across the layer, improving extraction compared to a purely diffusive scenario. To test the improved *E*_F_ alignment and reduced interface hole recombination velocity under actual working conditions, we implemented mono‐ and co‐doped TiO_2_ as an ESC into PSCs.

### 2.4 Photovoltaic Performance

**Figure 4:**
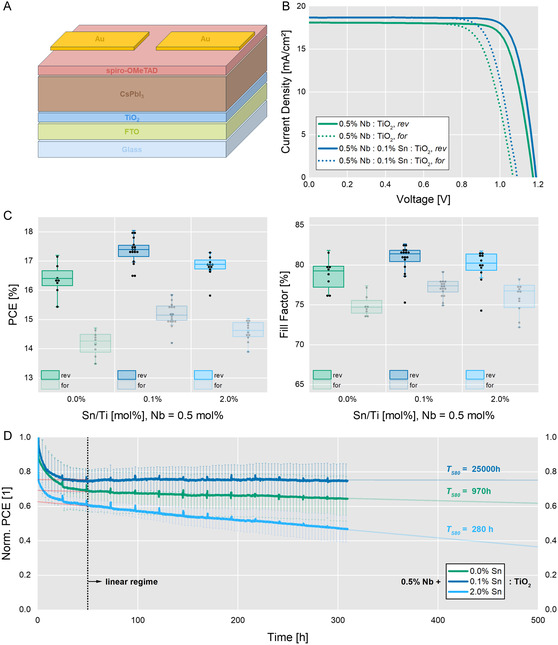
The co‐doping approach improves the photovoltaic parameters. A) The general device structure of the fabricated PSCs with Nb(V) and Sn(IV) present in TiO_2_. B) *J–V* characteristic of the best‐performing devices, showing increased *V*_OC_ and FF for the co‐doped sample. C) Statistical evaluation of the PSCs’ photovoltaic parameters shows significant improvement in PCE from 16.4% to 17.4%, mainly originating from FF improvement from 79% to 82%. D) Normalized device performance tested in MPP tracking under continuous AM1.5G illumination. The co‐doped champion does not exhibit significant performance loss over 300 h after the initial burn‐in phase. *T*_S80_ projections were obtained via linear regression of the MPP‐tracking data.

Our co‐doping strategy affects the WF of the ESC which, in turn, affects the charge carrier dynamics when contacted with CsPbI_3_. We implemented the developed (co‐)doped TiO_2_ layer in full photovoltaic devices, as shown in **Figure** **4**A, with spiro‐OMeTAD as HSC and gold as the back contact. The resulting *J–V* characteristic curves show, on average, an enhanced *V*_OC_ by 12 meV from 1.167 to 1.179 V (Figure S18, Supporting Information). Most evident, however, is the FF increase by 2.0% from 78.8% to 80.8%, as shown statistically in Figure 4C. These factors lead to a generally improved PCE by 1.0% from 16.4% to 17.4% (Figure 4C), with champion pixels of 17.2% for the mono‐doped TiO_2_ and 18.0% for the co‐doped TiO_2_ (Figure 4B). The recorded external quantum efficiency (EQE) spectra (Figure S19, Supporting Information) show a <5% deviation from the recorded *J–V* curves, therefore indicating accurate measurement.

Further, we assessed the stability of the PSCs via MPP tracking under 1 sun continuous illumination according to the ISOS‐L1‐I protocol.^[^ ^26^ ^]^ For comparability, Figure 4D shows the normalized performance of the PSCs; the absolute performances are shown in Figure S20, Supporting Information. All solar cells show a fast efficiency decay within the first 30 h, the so‐called burn‐in.^[^ ^27^ ^]^ This burn‐in phase is typical for device structures containing spiro‐OMeTAD and was associated with Li^+^ ion migration, which originates from the commonly used dopants in spiro‐OMeTAD.^[^ ^28^ ^]^ The end of the burn‐in phase is marked by the time *T*_S_ when the curve transitions into a linear regime which is set to 50 h for all traces in Figure 4D. In the average of around 10 pixels, co‐doped TiO_2_ outperforms the mono‐doped TiO_2_ as well as the sample over‐doped with 0.5% Nb(V) and 2.0% Sn(IV). Fitting the traces with linear regression starting at *T*_S_ = 50 h, as indicated in Figure 4D, yields degradation rates of –0.06 × 10^–4^, –1.50 × 10^–4^, and –5.24 × 10^–4^% percent per hour, respectively. From those slopes in the linear regime, we calculated the time at which the cells have degraded to 80% of the initial stabilized PCE (*T*_S80_),^[^ ^14^ ^]^ yielding projected *T*_S80_ lifetimes of around 25,000, 970, and 280 h (Figure S22, Supporting Information). Although those lifetimes are predictions, they exemplify the performance‐stabilizing effect of the optimized interface.

The linear degradation observed in our over‐doped TiO_2_ system suggests the continuous formation of deep, detrimental defects. In case of TiO_2_ co‐doped at optimum, the formation of those defects is suppressed. Based on ab initio density‐functional theory calculations, antisite defects, e.g., [2Cs]_Pb_, have been suggested as electronically active defects introducing deep trap levels under operating conditions in CsPbI_3_.^[^ ^29^ ^]^ We speculate that, by elevating the *E*_F_ in TiO_2_, the resulting change of chemical potentials inhibits the formation of a detrimental defect type. The improved stability is, therefore, a result of the optimized extractive and recombinative properties of the co‐doped TiO_2_ ESC.

## 3 Conclusion

This work focused on developing a strategy to enhance the stability of CsPbI_3_ PSCs by adjusting the surface trap‐state density and the WF of the TiO_2_ ESC through co‐doping with Nb(V) and Sn(IV). By combining structural and energetic modification of TiO_2_, we were able to improve the extraction of electrons from CsPbI_3_. By analyzing the trSPV curves of the two‐layered system, we identified the most effective extraction rate at co‐doping levels of 0.5% Nb(V) and 0.1% Sn(IV) in TiO_2_. Additionally, we introduced a method to fit trSPV curves using the DD model, which allowed us to extract fundamental parameters such as defect densities, interface recombination velocities, and extracted charge concentrations. Our findings indicated that using co‐doped TiO_2_ resulted in decreased interface hole recombination velocities and subsequently increased the concentration of extracted electrons. When implemented in complete photovoltaic devices, the co‐doped ESC performed better than the mono‐doped ESC in all relevant performance metrics. Additionally, incorporating 0.1% Sn(IV) increased the linearly projected *T*_S80_ lifetimes from 970 to 25,000 h, corresponding to a factor of 25. These results demonstrate that even a small compositional modification at the interface could lead to prolonged solid stabilization of the photovoltaic device.

## 4 Experimental Section

4.1

4.1.1

##### Solar Cell Fabrication

FTO glass substrates (*R*_S_ = 15 Ω sq^−1^, Yingkou Shangsheng Business Co. Ltd.) were cleaned via ultrasonication at 40 °C for 15 min each in a 2 vol% Mucasol (Schülke) solution in deionized (DI) water, followed by DI water, acetone (99.5%, Carl Roth), and *iso*‐propanol (IPA, 99.8%, Chemsolute). Prior to TiO_2_ deposition, substrates were treated with ultraviolet ozone (UV/O_3_) for 25 min at an O_2_ flow rate of 1.0 L min^−1^. For TiO_2_ deposition by spray pyrolysis, 15.15 mL of a 20.2 mM titanium(IV) bis(acetylacetonate) diisopropoxide [Ti(acac)_2_(O^i^Pr)_2_, 75 wt% in IPA, Sigma Aldrich] solution in ethanol (EtOH, 99.5%, Merck) was loaded into the spray nozzle connected to O_2_ as a carrier gas. Substrates were heated to 450 °C at 30 °C min^−1^ and sprayed in intervals of 10 s alternating with intervals of 30 s for film formation. After consumption of the precursor solution, substates were annealed at 450 °C for 1 h before the temperature was lowered to 150 °C. The substrates were used within 10 h or stored in air with reannealing to 450 °C for 1 h prior to further use. For Nb(V)‐doping of TiO_2_, a stock solution of NbCl_5_ (*c* = 10 mM, *M* = 270.17 g mol^−1^) in EtOH was prepared in a N_2_‐filled glove box. The doped Ti(acac)_2_(O^i^Pr)_2_ precursor solution was prepared as described earlier, replacing fractions of the added EtOH with the volumes of the dopant stock solution matching the desired atomic ratio. For Nb(V) and Sn(IV) co‐doping of TiO_2_, separate stock solutions of NbCl_5_ (as described earlier) and SnCl_4_ in EtOH (*c* = 10 mM, *M* = 260.52 g mol^−1^) were prepared. To prepare the perovskite ink, PbI_2_ (99.99%, TCI) was solubilized in *N*,*N*‐dimethylformamide (DMF, 99.8%, Sigma Aldrich) at 1.0 m and shaken overnight at 60 °C. A fraction of the PbI_2_ solution was added to CsI (99.999%, ABCR) powder in 1:1 mol mol^−1^ ratio and diluted with DMF to form a 0.7 m precursor ink. A part of the obtained precursor ink was added to dimethylammonium iodide (98%, Sigma Aldrich) powder in 1:1 mol mol^−1^ ratio relative to Cs. Separately, 45 mM methylammonium chloride (MACl, 99.99%, Dyenamo) solution in IPA (99.5%, Sigma Aldrich) and 3.0 mM n‐octylammonium iodide (OAI, >99%, GreatCell Solar) solution in IPA were prepared. Before perovskite deposition, the TiO_2_ substrates were treated with UV/O_3_ for 25 min. Substrates were preheated at 70 °C for 5 min in a N_2_ flow box. An amount of 80 μL of precursor ink were spin‐coated on the hot substrates at 3000 rpm for 30 s and 5000 rpm for 30 s, with 350 μL MACl solution dripped between steps. Films were transferred to a dry air glove box and annealed at 210 °C for 1:20 min. For posttreatment, perovskite films were treated with 200 μL of OAI solution in a N_2_‐filled glove box via spin‐coating at 5000 rpm for 30 s and annealing at 100 °C for 5 min. Stock solutions of 520 mg mL^−1^ lithium bis(trifluoromethanesulfonyl)imide (LiTFSI, 99.99%, Sigma Aldrich) in acetonitrile (ACN, 99.8%, Sigma Aldrich), and 375 mg mL^−1^ tris(2‐(1*H*‐pyrazol‐1‐yl)‐4‐*tert*‐butylpyridine)cobalt(III) tri[bis(trifluoromethane)‐sulfonamide] (FK209, >95%, Dyenamo) in ACN were prepared. Spiro‐OMeTAD (>99.8%, Lumtec) was dissolved in chlorobenzene (99.8%, Sigma Aldrich) to form a 90 mg mL^−1^ solution. To the spiro‐OMeTAD solution, 3.95 vol% 4‐*tert*‐butylpyridine (tBP, 98%, Sigma Aldrich), 2.30 vol% LiTFSI stock solution, and 1.00 vol% FK209 stock solution were added. The obtained solution was deposited on the posttreated perovskite films in a N_2_‐filled glove box via spin‐coating 80 μL spiro‐OMeTAD solution at 3500 rpm for 30 s. Substrates were then exposed overnight to O_2_ in dry air (RH = 0.1%). Finally, 100 nm of Au were thermally evaporated to form the top contact.

##### Characterization Methods

VASE was carried out on a Sentech SE 850 Deep Ultraviolet.

KPFM images were recorded in an argon‐filled glove box on an MFP3D microscope by Asylum Research. The Pt–Ir‐coated cantilever tip (Bruker SCM‐PIT, *f*_0_ = 75 kHz, *k* = 2.8 N m^−1^) with a nominal tip radius of 25 nm was calibrated against a freshly cleaved highly oriented pyrolytic graphite (HOPG) reference sample with a WF *ϕ* ^HOPG^ of (4.474 ± 0.005) eV.^[^ ^30^ ^]^ For the determination of CPD values, the histograms extracted from the (5 × 5) μm^2^ images were fitted with a Voigt function.

An ION‐TOF ToF‐SIMS V was utilized for depth profiling and chemical imaging of the TiO_2_ layers, utilizing methods covered in detail in previous reports.^[^ ^31^ ^]^ Analysis was completed utilizing a 3‐lens 30 keV BiMn primary ion gun. High‐mass‐resolution depth profiles were completed with a 30 keV Bi_3_^+^ primary ion beam (0.8 pA pulsed beam current), and a (50 × 50) μm^2^ area was analyzed with a 128:128 primary beam raster. The 3D tomography and high‐resolution imaging was completed with a 30 keV Bi_3_^++^ primary ion beam (0.1 pA pulsed beam current). Sputter depth profiling was accomplished with 1 keV cesium ion beam (6 nA sputter current).

HAXPES measurements were conducted at the high kinetic energy (HiKE) end station located at the BESSY II KMC‐1 beamline at Helmholtz–Zentrum Berlin für Materialien und Energie GmbH (HZB).^[^ ^32^ ^]^ The end station was equipped with a Scienta R4000 electron analyzer oriented perpendicular to the incoming X‐ray beam, with the polarization vector of the linearly polarized X‐rays aligned with the analyzer entrance. The measurements were performed using photons with an energy of 2 keV, provided by the KMC‐1 bending magnet beamline, employing the Si (111) crystal pair of the KMC‐1 double‐crystal monochromator (resulting in an experimental resolution of (0.30 ± 0.05) eV)^[^ ^33^ ^]^ in grazing incidence geometry, resulting in a (v × h) probing area of ≈100 μm × 3 mm. With 2 keV excitation, it was possible to probe the near‐surface bulk (roughly the top 10 nm with an exponentially decaying sensitivity) of a TiO_2_ sample.^[^ ^34^ ^]^ The energy scale of the HAXPES measurements was calibrated using Au 4f reference spectra of a clean Au foil, setting the BE of the Au 4f_7/2_ line to 84.00 eV. Curve fit analysis of the measured detail HAXPES spectra were simultaneously conducted with the Fityk software.^[^ ^35^ ^]^ Voigt profile functions and linear backgrounds were used for these fits. Spin–orbit doublets were fit using two Voigt functions with intensity ratios set to obey the 2*j* + 1 multiplicity rule. In contrast to the Nb 3d doublet peaks, the profile shape parameters of the Ti 2p doublet peaks were not constrained to be equal due to the inherent difference in width of their peaks (i.e., the 2p_1/2_ line showing a significantly wider peak than that of the 2p_3/2_ line, caused by a Coster–Kronig transition shortening the lifetime of Ti 2p_1/2_ core holes compared to those of Ti 2p_3/2_ core holes).^[^ ^36^ ^]^ HAXPES‐derived [Nb]:[Ti] composition ratio quantifications were carried out by correcting the peak intensities of the Nd 3d_5/2_ and Ti 2p_3/2_ core levels to account for differences in photoionization cross section,^[^ ^37^ ^]^ inelastic mean free path,^[^ ^34^ ^]^ and the transmission function of the electron analyzer.^[^ ^38^ ^]^

SEM was conducted on a Zeiss Merlin field‐emission scanning electron microscope with a Gemini II optical column. An accelerating voltage of 5 kV and a current of 100 pA were used. The images were recorded via the in‐lens detector.

XRD was carried out on a Bruker D8 diffractometer in Bragg–Brentano geometry, using Cu Kα radiation (*λ* = 1.5406 Å), 40 kV acceleration voltage, and 40 mA current. Samples were measured under inert conditions using airtight poly(methyl methacrylate) (PMMA) sample holders by Bruker.

Absolute steady‐state PL measurements on perovskite half‐cells were carried out in a N_2_‐filled glove box using a Quantum Yield Berlin (QYB) LuQY Pro prototype setup. The sample was illuminated from the substrate side by a laser (Insaneware) at 532 nm via a parabolic mirror. The laser power was 3.6 mW on an area of 0.14 cm^2^, resulting in a charge carrier density equivalent to ≈0.8 sun. The PL was collected with two plano‐convex lenses in series, and directed to a spectrometer (QE Pro, Ocean Insight) via an optical fiber. The data was processed by a software by QYB, using the generalized Planck law and the high‐energy tail method for estimation of the QFLS.^[^ ^39^ ^]^

The trPL measurements were performed on a confocal PL setup built in‐house. The setup featured a “80:20” “transmission:reflection” beam splitter to separate the excitation and detection path. A 700 nm diode laser (IB‐705‐B laser head with Taiko driver, Picoquant) with a pulse duration of around 100 ps and a repetition rate of 10 kHz were used for excitation. The laser beam was passed through a cleanup filter (FF01‐700/13–25, Semrock) and the power of the laser beam was tuned by a linear‐gradient neutral density filter to around 0.2 μW (≈0.8 μW readout on the power meter). An off‐axis parabolic mirror with 5 cm focal length was used for the focus and PL collection. The shape of the laser spot is a circle with around 250 μm diameter. A silicon single‐photon avalanche diode (Laser Components COUNT50) was employed for the PL detection. The signal was cut by a 715 nm long‐pass filter (FF01‐715/LP‐25, Semrock). The PL count and decay histogram were recorded by a TimeHarp260 Nano time‐correlated single‐photon counting module (Picoquant). The integration time was 600 s. All samples were encapsulated using thin cover glass and UV‐curable glue (BluFixx MGS Transparent) to protect the sensitive CsPbI_3_ films from air exposure.

trSPV measurements were carried out on a setup built in‐house on encapsulated samples. Laser excitation succeeded from the CsPbI_3_ surface at three different photon energies (1.8, 2.2, and 2.6 eV) from a tunable pumped pulse laser (Nd:YAG Laser, EKSPLA, NT230‐50‐SH/SF‐SCU‐2 H) at a pulse time of 3‐6 ns at a frequency of 2 Hz. Laser fluence was 15.0 nJ cm^−2^, controlled via neutral density filters. A total of 30 curves were recorded and averaged. The transients were measured with an oscilloscope card (Gage, CSE 1622‐ 4GS, 200 MS s^−1^) using an in‐house developed software for logarithmic readout.

Current density–voltage (*J*–*V*) curves were recorded at AM1.5G illumination on an Oriel LCS‐100 class ABB solar simulator in a N_2_‐filled glove box. The *J*–*V* curves shown were recorded at a step size of 0.02 V and integration and settling times of 50 ms.

EQE was measured on an Oriel Instruments QEPVSI‐B system, equipped with an SR810 DSP lock‐in amplifier, an Oriel Instruments 3502 optical chopper, and a Xe/HgXe lamp.

For long‐term MPP (LT‐MPP) tracking under continuous illumination, an MPP tracker built in‐house, equipped with a metal halide light source at 1 sun intensity and a UV filter, was used. The detailed setup description is published elsewhere by Köbler et al.^[30b]^

## Conflict of Interest

The authors declare no conflict of interest.

## Author Contributions

**Thomas W. Gries**: conceptualization (lead); formal analysis (lead); investigation (lead); project administration (lead); visualization (lead); and writing—original draft (lead). **Davide Regaldo**: formal analysis (lead); methodology (lead); resources (equal); software (lead); writing—original draft (supporting); and writing—review and editing (supporting). **Hans Köbler**: data curation (equal); formal analysis (equal); and investigation (supporting). **Noor Titan Putri Hartono**: data curation (equal); formal analysis (equal); and investigation (supporting). **Steven P. Harvey**: formal analysis (supporting); investigation (supporting); and visualization (supporting). **Maxim Simmonds**: formal analysis (supporting) and investigation (supporting). **Chiara Frasca**: formal analysis (supporting) and investigation (supporting). **Marlene Härtel**: resources (supporting). **Gennaro V. Sannino**: formal analysis (supporting); software (supporting); and validation (supporting). **Roberto Félix**: data curation (equal); formal analysis (equal); investigation (equal); visualization (supporting); and writing—original draft (supporting). **Elif Hüsam**: data curation (supporting); formal analysis (supporting); investigation (supporting); and visualization (supporting). **Ahmed Saleh**: data curation (supporting); formal analysis (supporting); investigation (supporting); and visualization (supporting). **Regan G. Wilks**: data curation (equal) formal analysis (equal); investigation (supporting); visualization (supporting); and writing—review and editing (supporting). **Fengshuo Zu**: formal analysis (supporting) and investigation (supporting). **Emilio Gutierrez-Partida**: formal analysis (supporting) and investigation (supporting). **Zafar Iqbal**: investigation (supporting). **Zahra Loghman Nia**: investigation (supporting). **Fengjiu Yang**: project administration (supporting). **Paola Delli Veneri**: supervision (supporting). **Kai Zhu**: supervision (supporting). **Martin Stolterfoht**: data curation (supporting); formal analysis (supporting); investigation (supporting); and writing—review and editing (supporting). **Marcus Bär**: data curation (equal); formal analysis (equal); resources (equal); and supervision (supporting). **Stefan A. Weber**: investigation (supporting); resources (equal); and supervision (supporting). **Philip Schulz**: resources (supporting) and software (supporting). **Jean‐Baptiste Puel**: resources (supporting); software (supporting); and writing—review and editing (supporting). **Jean‐Paul Kleider**: formal analysis (equal); methodology (equal); resources (equal); software (equal); supervision (supporting); and writing—review and editing (supporting). **Eva Unger**: conceptualization (supporting); supervision (supporting); and writing—review and editing (supporting). **Artem Musiienko**: conceptualization (lead); methodology (equal); project administration (equal); software (equal); supervision (lead); and writing—review and editing (equal). **Antonio Abate**: funding acquisition (lead); resources (lead); supervision (equal); and writing—review and editing (equal).

## Supporting information

Supplementary Material

## Data Availability

The data that support the findings of this study are available from the corresponding author upon reasonable request.
