# Supplementary material for: Co‐Doping Approach for Enhanced Electron Extraction to TiO2 for Stable Inorganic Perovskite Solar Cells
Source: Small Sci. 2025 May 6;5(7):2400578. doi: 10.1002/smsc.202400578 (PMC12257902; doi:10.1002/smsc.202400578)
Supplement: Supplementary file 1 — Supplementary Material [file SMSC-5-2400578-s001.pdf]

Supporting Information for

Co-Doping Approach for Enhanced Electron Extraction to TiO_2 for Stable Inorganic Perovskite Solar Cells

Thomas W. Gries, Davide Regaldo, Hans Köbler, Noor Titan Putri Hartono, Steven P. Harvey, Maxim Simmonds, Chiara Frasca, Marlene Härtel, Gennaro V. Sannino, Roberto Félix, Elif Hüsam, Ahmed Saleh, Regan G. Wilks, Fengshuo Zu, Emilio Gutierrez Partida, Zafar Iqbal, Zahra Loghman Nia, Fengjiu Yang, Paola Delli Veneri, Kai Zhu, Martin Stolterfoht, Marcus Bär, Stefan A. Weber, Philip Schulz, Jean-Baptiste Puel, Jean-Paul Kleider, Eva Unger, Qiong Wang, Artem Musiienko and Antonio Abate

S1 Measurement Methods

Variable-angle spectroscopic ellipsometry (VASE) was carried out on a Sentech SE 850 DUV.

Kelvin-probe force microscopy (KPFM) images were recorded in an argon-filled glovebox on an MFP3D microscope by Asylum Research. The Pt-Ir-coated cantilever tip (Bruker SCM-PIT, f_0 = 75 kHz, k = 2.8 N m^{-1}) with a nominal tip radius of 25 nm was calibrated against a freshly cleaved highly oriented pyrolytic graphite (HOPG) reference sample with a work function (WF) ϕ^{HOPG} of (4.474 ± 0.005) eV.[1] For the determination of contact potential difference (CPD) values, the histograms extracted from the (5 x 5) μm^2 images were fitted with a Voigt function. The peak maximum was taken as the average value with the full width at half maximum (FWHM) of the curve being the uncertainty. Calculation of the absolute work function (WF) is described in **Sec. S3.2**.

An ION-TOF TOF-SIMS V Time of Flight SIMS (ToF-SIMS) spectrometer was utilized for depth profiling and chemical imaging of the TiO_2 layers, utilizing methods covered in detail in previous reports.[2] Analysis was completed utilizing a 3-lens 30 keV BiMn primary ion gun. High mass resolution depth profiles were completed with a 30 keV Bi_3^+ primary ion beam, (0.8 pA pulsed beam current), a (50 x 50) μm^2 area was analyzed with a 128:128 primary beam raster. 3D tomography and high-resolution imaging was completed with a 30 keV Bi_3^{++} primary ion beam, (0.1 pA pulsed beam current). Sputter depth profiling was accomplished with 1 keV cesium ion beam (6 nA sputter current).

Hard X-ray photoelectron spectroscopy (HAXPES) measurements were conducted at the High Kinetic Energy (HiKE) endstation located at the BESSY II KMC-1 beamline at Helmholtz-Zentrum Berlin für Materialien und Energie GmbH (HZB).[3] The endstation is equipped with a Scienta R4000 electron analyzer oriented perpendicular to the incoming X-ray beam, with the polarization vector of the linearly polarized X-rays aligned with the analyzer entrance. The

measurements were performed using photons with an energy of 2 keV, provided by the KMC-1 bending magnet beamline, employing the Si (111) crystal pair of the KMC-1 double crystal monochromator [resulting in an experimental resolution of (0.30 ± 0.05) eV][4] in grazing incidence geometry, resulting in an (v × h) probing area of approximately 100 μm × 3 mm. With 2 keV excitation, it is possible to probe the near-surface bulk (roughly the top 10 nm with an exponentially decaying sensitivity) of a TiO_2 sample.[5] The energy scale of the HAXPES measurements was calibrated using Au 4f reference spectra of a clean Au foil, setting the binding energy (BE) of the Au $4f_{7/2}$ line to 84.00 eV. Curve fit analysis of the measured detail HAXPES spectra were simultaneously conducted with the Fityk software.[6] Voigt profile functions and linear backgrounds were used for these fits. Spin-orbit doublets were fit using two Voigt functions with intensity ratios set to obey the $2j+1$ multiplicity rule. In contrast to the Nb 3d doublet peaks, the profile shape parameters of the Ti 2p doublet peaks were not constrained to be equal due to the inherent difference in width of their peaks (i.e., the $2p_{1/2}$ line showing a significantly wider peak than that of the $2p_{3/2}$ line, caused by a Coster-Kronig transition shortening the lifetime of Ti $2p_{1/2}$ core holes compared to those of Ti $2p_{3/2}$ core holes).[7] HAXPES-derived [Nb]:[Ti] composition ratio quantifications were carried out by correcting the peak intensities of the Nd $3d_{5/2}$ and Ti $2p_{3/2}$ core levels to account for differences in photoionization cross section,[8] inelastic mean free path (IMFP)[5] and the transmission function of the electron analyzer.[9]

Scanning electron microscopy (SEM) was conducted on a Zeiss Merlin field emission scanning electron microscope with a Gemini II optical column. An accelerating voltage of 5 kV and a current of 100 pA were used. The images were recorded via the in-lens detector.

X-ray diffraction (XRD) was carried out on a Bruker D8 diffractometer in Bragg-Brentano geometry, using Cu Kα radiation (λ = 1.5406 Å), 40 kV acceleration voltage and 40 mA current. Samples were measured under inert conditions using airtight PMMA sample holders by Bruker.

Absolute steady-state photoluminescence (PL) measurements on perovskite half-cells were carried out in a nitrogen-filled glovebox using a Quantum Yield Berlin (QYB) LuQY Pro prototype setup. The sample was illuminated from the substrate side by a laser (Insaneware) at 532 nm via a parabolic mirror. The laser power was 3.6 mW on an area of 0.14 cm^2, resulting in a charge carrier density equivalent to approximately 0.8 sun. The PL was collected with two plano-convex lenses in series, and directed to a spectrometer (QE Pro, Ocean Insight) via an optical fiber. The data was processed by a software by QYB, using the generalized Planck law and the high-energy tail method for estimation of the quasi-Fermi level splitting (QFLS).[10]

Transient PL measurements were performed on a confocal PL setup built in-house. The setup featured a “80:20”- “transmission:reflection”-beam splitter to separate the excitation- and detection-path. A 700 nm diode laser (IB-705-B laser head with Taiko driver, Picoquant) with a pulse duration of around 100 ps and a repetition rate of 10 kHz were used for excitation. The laser beam was passed through a cleanup filter (FF01-700/13–25, Semrock) and the power of

the laser beam was tuned by a linear-gradient neutral density filter to around 0.2 μW (≈ 0.8 μW readout on the power meter). An off-axis parabolic mirror with 5 cm focal length was used for the focus and PL collection. The shape of the laser spot is a circle with around 250 μm diameter. A silicon single-photon avalanche diode (Laser Components COUNT50) was employed for the PL detection. The signal was cut by a 715 nm long-pass filter (FF01-715/LP-25, Semrock). The PL count and decay histogram were recorded by a TimeHarp260 Nano time-correlated single photon counting module (Picoquant). The integration time was 600 s. All samples were encapsulated using thin cover glass and UV-curable glue (BluFixx MGS Transparent) to protect the sensitive $CsPbI_3$ films from air exposure.

Transient surface photovoltage (trSPV) measurements were carried out on a setup built in-house on encapsulated samples. Laser excitation succeeded from the $CsPbI_3$ surface at three different photon energies (1.8 eV, 2.2 eV and 2.6 eV) from a tunable pumped pulse laser (Nd:YAG Laser, EKSPLA, NT230-50-SH/SF-SCU-2H) at a pulse time of 3-6 ns at a frequency of 2 Hz. Laser fluence was 15.0 nJ cm^{-2}, controlled via neutral density filters. A total of 30 curves were recorded and averaged. The transients were measured with an oscilloscope card (Gage, CSE 1622- 4GS, 200 MS s^{-1}) using an in-house developed software for logarithmic read-out.

Current-Voltage (*J-V*) curves were recorded at AM1.5G illumination on an Oriel LCS-100 class ABB solar simulator in a nitrogen-filled glovebox. The *J-V* curves shown were recorded at a step size of 0.02 V and integration and settling times of 50 ms.

External quantum efficiency (EQE) was measured on an Oriel Instruments QEPVSI-B system, equipped with a SR810 DSP lock-in amplifier, an Oriel Instruments 3502 optical chopper and a xenon/mercury-xenon lamp.

For long-term maximum power point (LT-MPP) tracking under continuous illumination, an MPP-tracker built in-house, equipped with a metal halide light source at 1 sun intensity and a UV-filter, was used. The detailed setup description is published elsewhere by Köbler et al.[11]

S2 Solar Cell Preparation

S2.1 Materials

Titanium(IV) bis(acetylacetonate) diisopropoxide ($Ti(acac)_2(O^iPr)_2$, 75 wt% in 2-propanol, Sigma Aldrich), dimethylammonium iodide (DMAI, 98%, Sigma Aldrich), cesium iodide (CsI, 99.999%, ABCR), lead(II) iodide (PbI_2, 99.99%, TCI), methylammonium chloride (MACl, 99.99%, Dyenamo), *n*-octylammonium iodide (OAI, >99%, GreatCell Solar), 2,2',7,7'-Tetrakis(*N*,*N*-di-p-methoxyphenylamino)-9,9'-spirobifluorene (spiro-OMeTAD, >99.8%, Lumtec), tris(2-(1*H*-pyrazol-1-yl)-4-*tert*-butylpyridine)cobalt(III)tri[bis(trifluoromethane)-sulfonimide] (FK209, >95%, Dyenamo), lithium bis(trifluoromethanesulfonyl)imide (LiTFSI, 99.99%, Sigma Aldrich), 4-*tert*-butylpyridine (*t*BP, 98%, Sigma Aldrich), *N*,*N*-dimethylformamide (DMF, 99.8%, Sigma Aldrich), 2-propanol (IPA, 99.5%, Sigma Aldrich), chlorobenzene (CBZ, 99.8%, Sigma Aldrich), acetonitrile (ACN, 99.8%, Sigma Aldrich), ethanol (EtOH, 99.5%, Merck).

S2.2 Sample Cleaning

For the fabrication of inorganic perovskite solar cells, fluorine doped tin oxide (FTO) covered glass substrates ($R_S = 15\ \Omega\ sq^{-1}$) were used. The FTO substrates are laser-etched on two opposite edges by the manufacturer. The surface was wettened with tap water and subsequently brushed using a solution of Mucasol (Schülke) in deionized (DI) water (2% $v\ v^{-1}$). The substrates were then immersed in fresh Mucasol solution and subjected to ultrasonication (EMAG Emmi-40HC) at 40 °C for 15 min before washing with DI-water five times. Afterwards, the substrates were immersed in DI-water, again ultrasonicated for 15 min and washed once with acetone. After another ultrasonication step in acetone for 15 min, the solvent was exchanged to *iso*-propanol (IPA) for the last ultrasonication for 15 min. The clean substrates were either used directly or were stored under IPA overnight.

S2.3 Titanium Dioxide Layer

Prior to the deposition of the TiO_2 layer, the cleaned substrates, blow-dried with nitrogen, were subjected to 25 min of ultraviolet ozone (UV/O_3) treatment (FHR UVOH 150 Lab) at an oxygen flow rate of 1.0 L min^{-1}. For the layer deposition via spray pyrolysis, 15.15 mL of a 20.2 mM precursor solution of $Ti(acac)_2(O^iPr)_2$ in EtOH were prepared in a vial before being transferred into the reservoir. Oxygen was used as a carrier gas. The pristine substrates were positioned on a high-temperature hot plate and heated to 450 °C at a rate of 30 °C min^{-1}. After a stabilization period of 10 min at 450 °C, the spray coating was started. Here, spraying intervals of 10 s alternated with evaporation intervals of 30 s. After consumption of the prepared precursor solution, the substates were annealed at 450 °C for 1 h before the temperature was down-regulated to 150 °C at a rate of 60 °C min^{-1} and kept at that temperature for 10 h. The substrates, now coated with a 20 nm thick layer of TiO_2, were either used within the period of 10 h for subsequent depositions or stored in air. In case of storage, the substrates

were re-heated to 450 °C for 1 h before being used in further steps. For doping the TiO_2 layer with niobium, a stock solution of niobium(V) chloride (c = 10 mM, M = 270.17 g mol^{-1}) in EtOH was prepared in a nitrogen-filled glovebox. The doped $Ti(acac)_2(O^iPr)_2$ precursor solution was prepared as described above, replacing fractions of the added EtOH with the volumes of the dopant stock solution matching the desired atomic ratio.

For co-doping the TiO_2 layer both with niobium and tin, separate stock solutions of niobium(V) chloride, as described above, and tin(IV) chloride in EtOH (c = 10 mM, M = 260.52 g mol^{-1}) were prepared and processed in an equal way.

S2.4 Perovskite Layer

PbI_2, CsI and DMAI were scaled on a high-precision balance (Kern ABT 120-5DNM, d = 0.01 mg) in a solvent-free, nitrogen-filled glovebox (mBraun MB200B, H_2O = 0.1 ppm, O_2 = 0.1 ppm) and then transferred to a different glovebox for dissolution in the respective solvents. DMF was added to PbI_2 to form a 1.0 M solution, which was shaken overnight at 60 °C (CellMedia TS basic). A fraction of the PbI_2 solution was then added to CsI powder in a ratio of 1:1 mol/mol and diluted with DMF to form a 0.7 M precursor ink. Subsequently, a part of the obtained precursor ink was added to DMAI powder in a 1:1 molar ratio with respect to Cs. Separately, a 45 mM solution of MACl in IPA and a 3.0 mM solution of OAI in IPA were prepared. Prior to perovskite deposition, the TiO_2-coated substrates were subjected to UV/O_3 treatment at an oxygen flow rate of 1.0 L min^{-1} for 25 min. The deposition was then carried out via spin-coating in a nitrogen-filled flowbox (mBraun MB LaminarFlow, H_2O = 0.1 ppm, O_2 = 0.1 ppm). Substrates were preheated to 70 °C on a hot plate for 5 min and quickly transferred to the spin-coater. 80 µL of the precursor ink were extruded onto the hot substrate before the spin-coating program was started. The program consisted of two steps at 3000 rpm for 30 s and 5000 rpm for 30 s, separated by a halt of 5 s. During the halt, 350 µL of MACl solution were dispensed over the surface. After deposition, the substrates were quickly transferred to a glovebox filled with dry air, where the annealing step was carried out on a hot plate at 210 °C for 1:20 min.

S2.5 Post-Annealing Treatment

For subsequent treatment of the dark perovskite films, the substrates were transferred to a nitrogen-filled glovebox (mBraun MB LaminarFlow, H_2O = 0.1 ppm, O_2 = 0.1 ppm). The substrates were placed on the spin-coater and 200 µL of OAI solution were dropped onto the surface. After self-spreading of the solution over the surface (~ 5 s), spinning was started at 5000 rpm with an acceleration of 1000 rpm s^{-1}. Subsequently, the substrates were annealed on a hotplate at 100 °C for 5 min.

S2.6 Spiro-OMeTAD Layer

In a nitrogen-filled glovebox, stock solutions of 520 mg mL^{-1} LiTFSI in ACN and 375 mg mL^{-1} FK209 in ACN were prepared. Subsequently, spiro-OMeTAD was dissolved in CBZ to form a 90 mg mL^{-1} solution. To the obtained solution 3.95 vol% *t*BP, 2.30 vol% LiTFSI stock solution and 1.00 vol% FK209 stock solution were added, each with respect to the initial volume of the spiro-OMeTAD solution. The deposition of the spiro-OMeTAD layer was carried out in a nitrogen-filled glovebox. The post-treated substrates were cleaned with a nitrogen-gun and placed on the spin-coater. 80 µL of the spiro-OMeTAD solution were spread across the entire surface with a pipette tip before spinning at 3500 rpm with an acceleration of 700 rpm s^{-1} was started. After deposition, the substrates were exposed to O_2 in a storage box filled with dry air (RH = 0.1%).

S2.7 Gold Electrode

Prior to the evaporation of the gold electrodes, the deposited layers were removed with a scalpel within an approximately 4 mm broad strip on one edge bridging the two FTO-free edges on the substrate. After cleaning the dust with a nitrogen-gun, the substrates were placed with 0.18 cm^2 masks on top of the spiro-OMeTAD layer in a glovebox-integrated evaporator. A 100 nm thick layer was thermally evaporated according to the rate program specified in **Tab. S1**.

Tab. S1. Rate program for the evaporation of gold on top of the solar cell.

Rate [kÅ s^{-1}]	Thickness Range [kÅ]
0.08	0.00 – 0.01
0.10	0.01 – 0.02
0.20	0.02 – 0.05
0.50	0.05 – 0.20
1.00	0.20 – 1.00

S2.8 Sputtering of Indium Zinc Oxide

The indium zinc oxide (IZO) films were deposited using magnetron sputtering at the Von Ardenne sputtering module of the *Multifunctional Cluster Tool for the Production of Perovskite-Based Tandem Solar Cells* (*KOALA*). A ceramic IZO tube target of 0.6 m, composed of 90 wt.% In_2O_3 and 10 wt.% ZnO (Advanced Nano Products) was utilized for the thin film sputter deposition. The sputtering process was conducted at a direct current (DC) excitation power of 2 kW, a process pressure of approximately 4 µbar, and a target-to-substrate distance of 160 mm, without intentional heating. The sputtering gas consisted of argon with 2% oxygen.

S3 Measurement Details

S3.1 VASE Measurements

For estimation of TiO_2 layer thickness, different volumes of either 20.2 M or 2.02 M were sprayed on different areas of cleaned quartz glass substrates. The resultant layer thickness was then determined via fitting of VASE data. Normalization of the total sprayed amount of n[Ti(acac)$_2$(O^{i}Pr)$_2$] in mmol to the coated area A in units sq, implies a root-function-like growth mechanism. At the condition used for solar cells [*i.e.* 15.15 mL of a 20.2 mM Ti(acac)$_2$(O^{i}Pr)$_2$ solution per 16 FTO substrates] and characterizations as well as simulations, constant 20 nm were assumed as reproducible layer thickness.

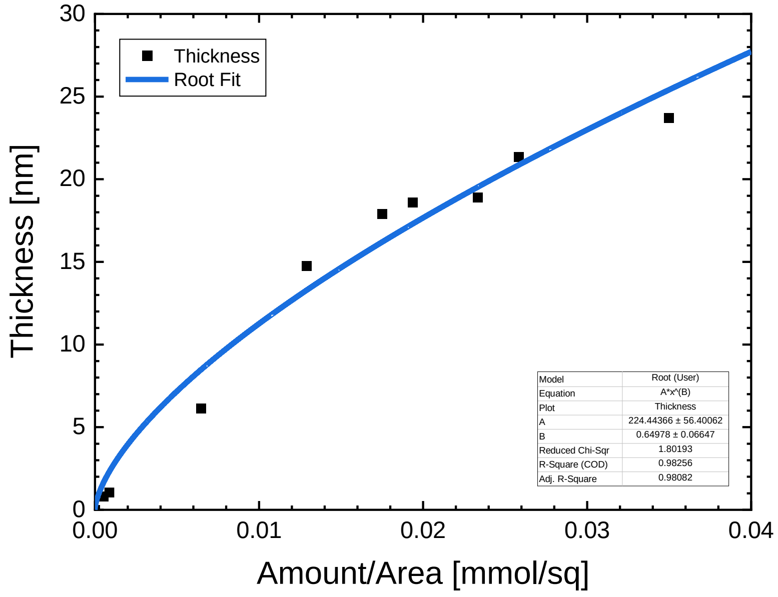

Fig. S1. Growth of the TiO_2 layer during the spray pyrolysis process. The sprayed amount of Ti(acac)$_2$(O^{i}Pr)$_2$ precursor was normalized to the coated area. The values were obtained via VASE.

S3.2 KPFM Measurements

All KPFM scans were recorded in top-view with one and the same cantilever. The cantilever was calibrated against HOPG in the beginning and in the end of the measurement series. To show reproducibility of the obtained CPD values, at least two images were recorded at different positions of each sample. The entire series is given in **Fig. S2**. Calibration of the cantilever against HOPG showed a drift of around 20 mV comparing the beginning and the end of the series, associated with tip abrasion or debris adhesion. We assumed this process to be linear, leading to the linear baseline in red in **Fig. S2**.

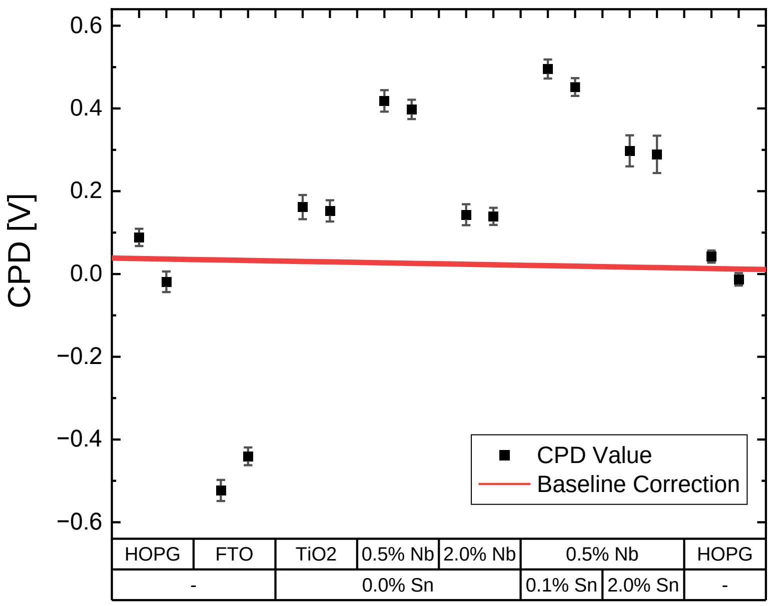

Fig. S2. The KPFM-scan series elucidates different CPD between the sample and the cantilever tip. A linear baseline was assumed between the initial and final calibration of the cantilever, accounting for gradual degradation. Displayed data points are the maxima of Voigt fits of the image histograms, while error bars represent the FWHM.

The obtained raw CPD values of **Fig. S2** were recalculated according to baseline levelization (s. **Fig. S3**). Reliability of the sample material is expressed in the very small deviation of obtained values recorded in different sample regions.

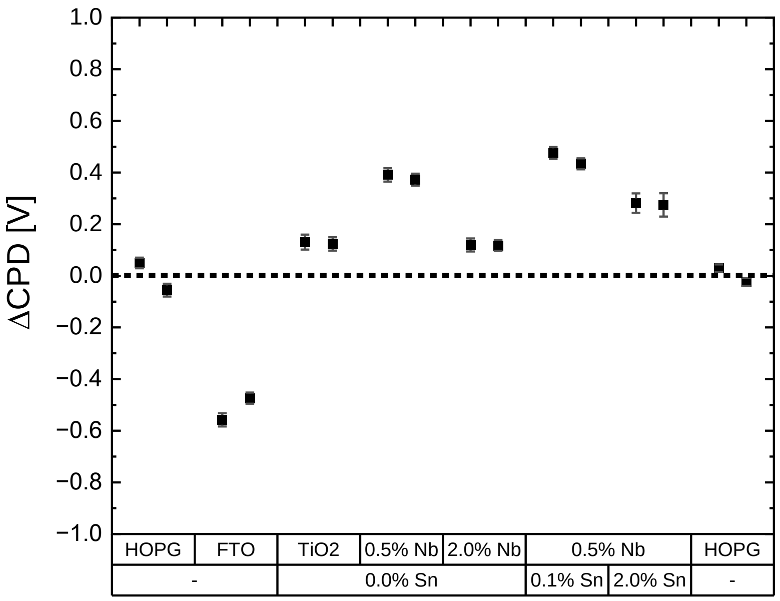

Fig. S3. Obtained CPD values after baseline levelization. All measurements show good reproducibility in different sample regions.

The levelized ΔCPD values were then linearly transformed to WF via **Eq. S1** and **Eq. S2**, where ϕ^{HOPG} is known to be (4.474 ± 0.005) eV[1] and e represents unit charge.

(Eq. S1) $$\phi^{\mathrm{tip}} = e\, U_{CPD}{}^{HOPG} + \phi^{\mathrm{HOPG}}$$

(Eq. S2) $$\phi^{\mathrm{sample}} = \phi^{\mathrm{tip}} - e\, U_{CPD}{}^{sample}$$

Transformed Voigt fits are displayed in **Fig. S4**, and in **Fig. 1** in the main text. Extracted values are listed in **Tab. S2** below.

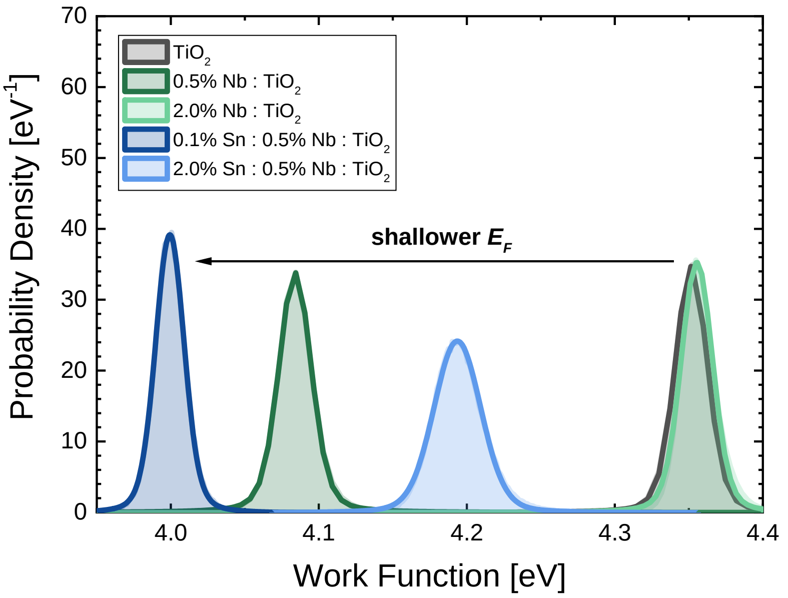

Fig. S4. Both Nb(V) and Sn(IV) dopant series exhibit minima in WF. These minima are found at 0.5% Nb(V) and 0.1% Sn(IV) with respect to TiO_2. Adding dopant concentrations beyond those optima leads to a reversal of the trend, and WF increases again.

Tab. S2. Sample WF (ϕ^{sample}) depending on dopant concentration in TiO_2 extracted from KPFM.

Composition Host Material	Nb(V)/Ti(IV) [mol%]	Sn(IV)/Ti(IV) [mol%]	ϕ^{sample} [eV]
TiO_2	0	0	(4.35 ± 0.03)
TiO_2	0.5	0	(4.08 ± 0.03)
TiO_2	2.0	0	(4.36 ± 0.03)
TiO_2	0.5	0.1	(4.00 ± 0.02)
TiO_2	0.5	2.0	(4.20 ± 0.04)

Similarly, the WF of $CsPbI_3$ was determined via KPFM scan of a (5 × 5) µm^2 sample area. The topography and CPD images are shown in **Fig. S5A** and **Fig. S5B**. Transformation of the CPD histogram to WF succeeded according to **Eq. S1** and **Eq. S2**, assuming ϕ^{HOPG} = (4.474 ± 0.005) eV.[1]

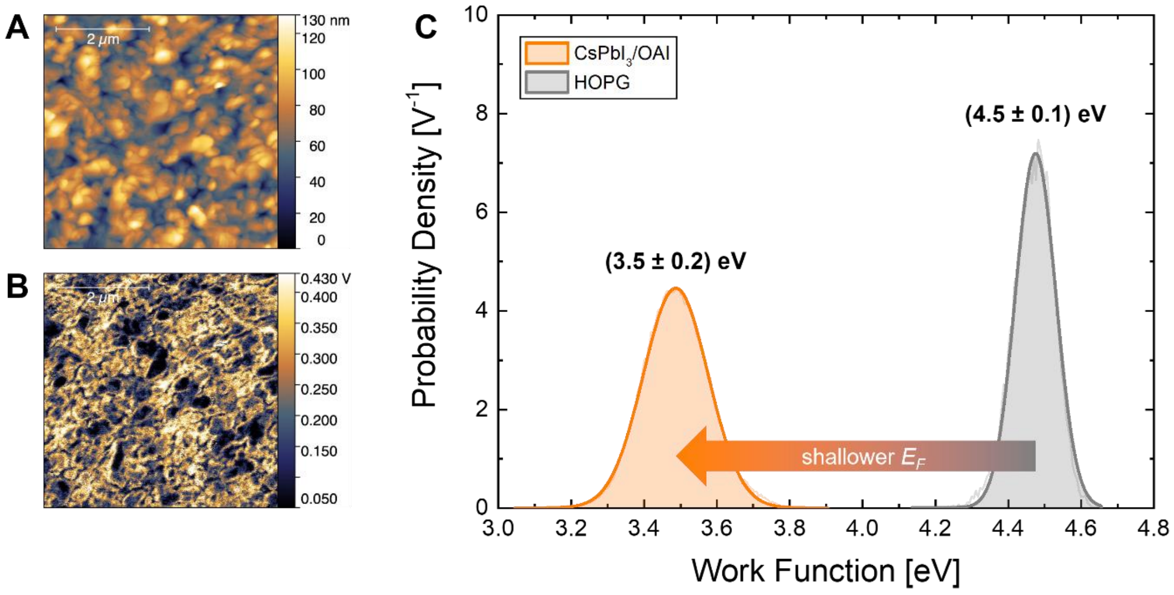

Fig. S5. **(A)** Topography scan of a (5 x 5) μm^2 area of OAI-treated $CsPbI_3$, exhibiting a maximum height of 130 nm with an RMS roughness of 17 nm. **(B)** CPD channel of the $CsPbI_3$ area shown in (A) with an average CPD of (0.2 ± 0.1) eV. **(C)** Histogram of the CPD values obtained from (B) transformed to WF values (orange) via referencing to HOPG (gray).

S3.3 ToF-SIMS Measurements of (doped) TiO_2

The elemental distribution of Sn(IV) and Nb(V) was determined via ToF-SIMS. According to **Sec. S2.3**, samples of pristine TiO_2 (non-doped), 0.5% Nb(V) doped TiO_2 (mono-doped), and 0.1% Sn and 0.5% Nb doped TiO_2 (co-doped) were fabricated on FTO substrates. Since quantification of the low amount of Sn dopant is not possible on Sn-containing FTO, we additionally prepared the sample series on IZO. IZO substrates were fabricated in-house via magnetron DC sputtering, as described in **Sec. S2.8**.

Lateral dopant homogeneity was confirmed either via non-bunched mode for high resolution imaging, or bunched mode for spectrometric imaging. Obtained images of Nb-, Sn-, Ti-, and F-related signals of the sample series on FTO are shown in **Fig. S6**. For the distinction of the Sn(IV) dopant contribution and the FTO-substrate contribution, images were recorded on the same sample series fabricated on IZO (Fig. S7). For detection of Nb, the $^{93}Nb^+$ mass peak was used. The detection of Sn succeeded via summation of the counts of the Sn^+ isotope pattern, including $^{112}Sn^+$, $^{114}Sn^+$, $^{115}Sn^+$, $^{116}Sn^+$, $^{117}Sn^+$, $^{118}Sn^+$, $^{119}Sn^+$, $^{120}Sn^+$, $^{122}Sn^+$, and $^{124}Sn^+$. For the detection of the host matrix element Ti, the lower-abundant isotope peaks of $^{46}Ti^+$, $^{47}Ti^+$, $^{49}Ti^+$, and $^{50}Ti^+$ were used for sensitivity reasons. Additionally, the $^{19}F^+$ mass peak was used for the direct detection of substrate-related signals. The detected signals do not directly reflect the stoichiometric ratio due to the SIMS ionization probabilities differing between the elements.

Sn(IV)- and Nb(V)- dopants are distributed homogeneously over the tested (50 × 50) μm^2 surface area, without indication of lateral agglomeration of either element (**Fig. S6**). Further, the low magnitude of F-related signal suggests pinhole-free formation of TiO_2 on FTO.

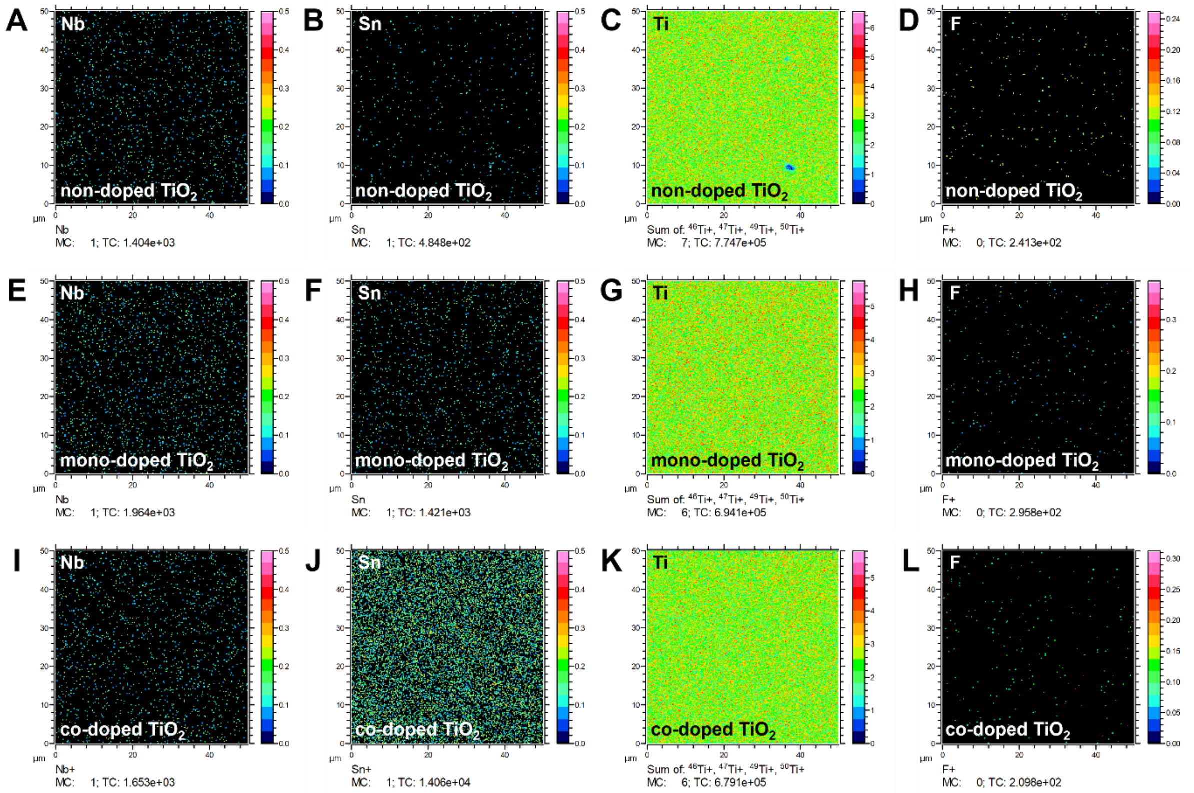

Fig. S6. **The lateral distribution of Sn(IV) and Nb(V) dopants in TiO_2 on FTO is homogeneous across the probed area of (50 × 50) μm^2. (A, B)** The Nb- and Sn- related signals in non-doped TiO_2 represent the FTO-convoluted background levels of the elements on the TiO_2 surface. **(C, D)** The homogeneity of the Ti- and the low magnitude of the F-related signals show no tendency of non-doped TiO_2 for pinhole formation. **(E, F)** The Nb-related signal at the sample surface is doubled for mono-doped TiO_2, while also the Sn-related signal increases. The unexpected increase of the Sn-related signal may be related to Sn-migration from the FTO-substrate. **(G, H)** The Ti-related signal remains homogeneous, and the F-related signal remains low in mono-doped TiO_2, confirming pinhole-free formation. **(I, J)** The Nb-related signal at the surface is comparable to mono-doped TiO_2, while Sn-related signal at the surface increases by one order of magnitude in co-doped TiO_2. Both distributions are homogeneous. **(K, L)** The Ti-related signal is homogeneous, and the F-related signal remains low in co-doped TiO_2, further confirming pinhole-free formation.

To remove any signal contribution of Sn in FTO, images of Nb-, Sn-, and Ti-related signals were recorded on the same sample series on IZO substrates instead of FTO substrates (Fig. S7). Again, lateral dopant homogeneity across the probed area of (300 × 300) μm^2 is confirmed by the uniform Nb- and Sn-related distributions. Further, the Ti-related signal indicates pinhole-free formation of TiO_2 on IZO.

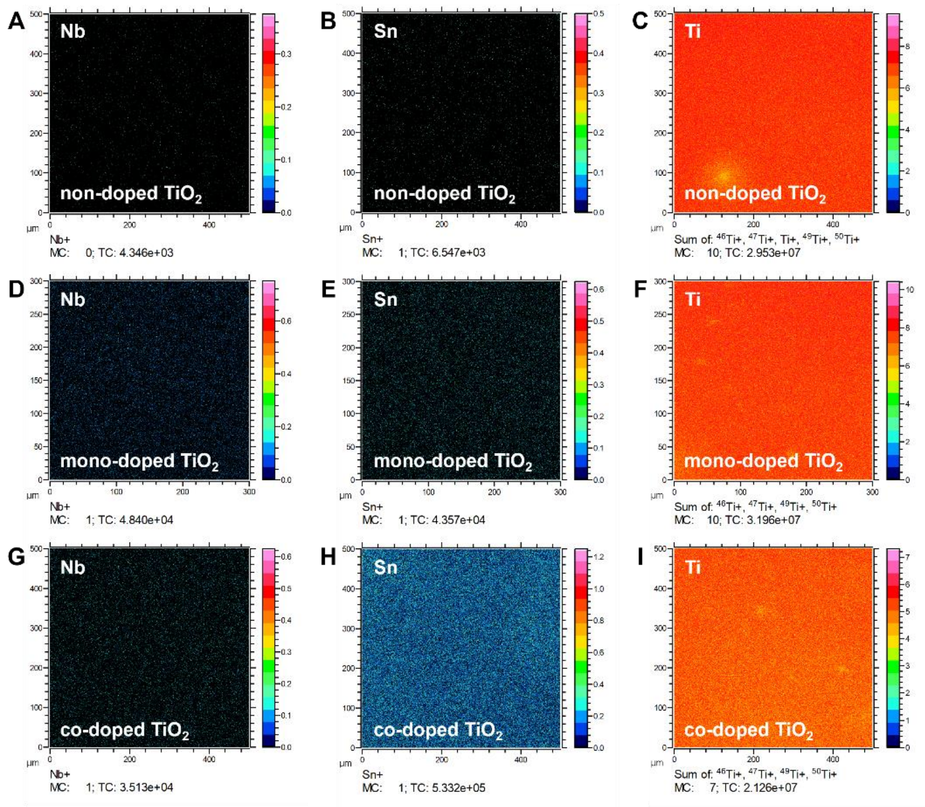

Fig. S7. **The lateral distribution of Sn(IV) and Nb(V) dopants in TiO_2 on IZO is homogeneous across the probed area of (300 × 300) μm^2. (A, B**) The Nb- and Sn- related signals in non-doped TiO_2 represent the background levels of the elements on the TiO_2 surface. **(C)** The homogeneity of the Ti-related signal shows no tendency of non-doped TiO_2 for pinhole formation. **(D, E)** The Nb-related signal at the sample surface increases by one order of magnitude for mono-doped TiO_2. **(F)** The Ti-related signal remains homogeneous, confirming pinhole-free formation. **(G, H)** The Nb-related signal at the surface is comparable to mono-doped TiO_2, while Sn-related signal at the surface increases by one order of magnitude in co-doped TiO_2. Both distributions are homogeneous. **(I)** The Ti-related signal is homogeneous, further confirming pinhole-free formation.

We further recorded the vertical Sn(IV) and Nb(V) dopant distributions via ToF-SIMS depth profiling in bunched mode. The depth-resolved occurrence of Nb-, Sn-, Ti-, and F-elements are shown separately for the sample series on FTO in **Fig. S8A** to **Fig. S8C**, and for the sample series on IZO in **Fig. S8D** to **Fig. S8F**. A layer thickness of approximately 20 nm was assumed based on the ellipsometry measurements presented in **Fig. S1**.

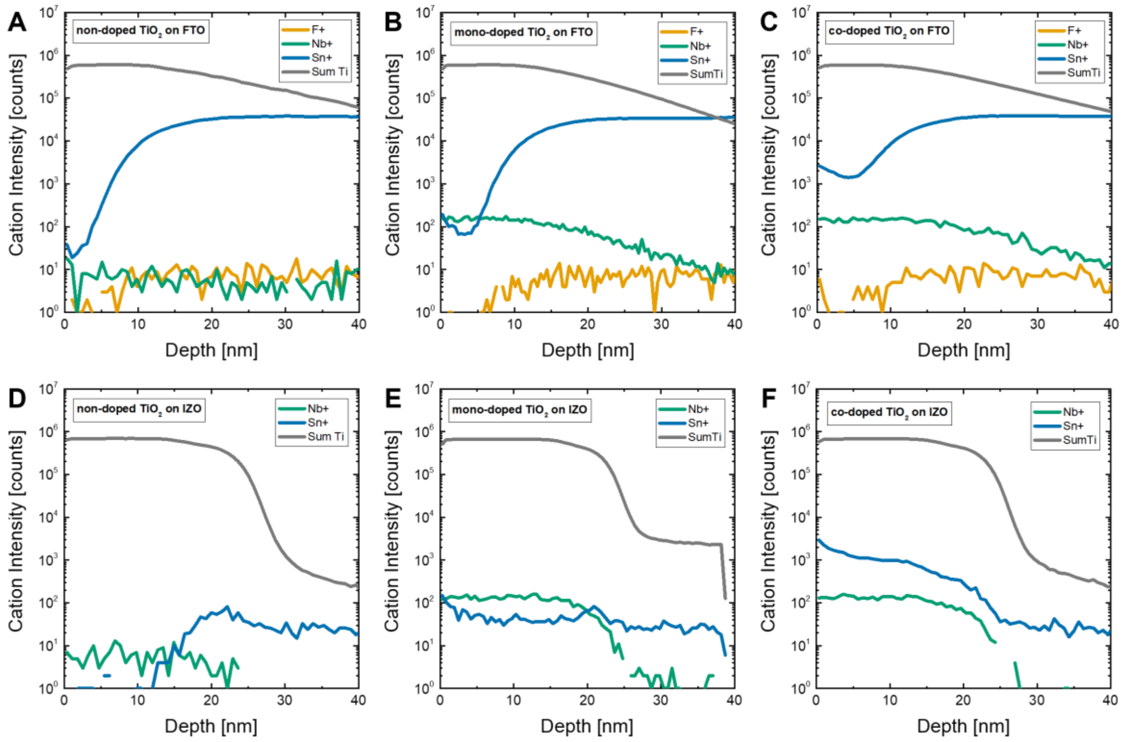

Fig. S8. The vertical element distributions of doped TiO_2 layers were recorded via ToF-SIMS depth profiling. The first row shows F-, Nb-, Sn-, and Ti-related signals of **(A)** non-doped TiO_2, **(B)** mono-doped TiO_2, and **(C)** co-doped TiO_2 deposited on FTO. The second row shows Nb-, Sn-, and Ti-related signals of **(D)** non-doped TiO_2, **(E)** mono-doped TiO_2, and **(F)** co-doped TiO_2 deposited on IZO.

To resolve the vertical distribution of Sn(IV) and Nb(V) dopants in TiO_2, and to distinguish the Sn-signal contributions of the dopant from the FTO substrate in co-doped TiO_2, we compared the Nb- and Sn-related curves from non-, mono-, and co-doped TiO_2 on FTO (**Fig. S8A** and **Fig. S8C**) and non-, mono- and co-doped TiO_2 on IZO (**Fig. S8D** and **Fig. S8E**). For better comparability, the curves were normalized to the respective maximum signal, which originated from the host matrix element Ti in all cases.

The normalized curves of Nb-related signal in non-, mono-, and co-doped TiO_2 are presented in logarithmic scale in **Fig. S9A** and in linear scale in **Fig. S9B**. The profiles of mono- and co-doped confirm homogeneous vertical distribution and, therefore, support the hypothesis of full Nb(V) integration into the TiO_2 crystal structure.

To elucidate the depth distribution of Sn dopant, we compared the Sn-related signal of non-doped TiO_2 on IZO (S_0) and on FTO (S_1), and of co-doped TiO_2 on IZO (S_2) and on FTO (S_3). S_0 represents the background level of Sn in a Sn-free sample. S_1 is the Sn-signal contribution in non-doped TiO_2, purely originating from the FTO substrate. S_2 represents the Sn-signal contribution purely from the dopant in co-doped TiO_2 on IZO. S_3 is the convolution of Sn-signal from the dopant, from the FTO substrate, and from the background, and should ideally be the result of the addition of S_0, S_1, and S_2.

Fig. S9C shows the normalized curves of S_0 to S_3 on a logarithmic scale. The black dotted curve represents the numeric addition of the signal components S_0, S_1 and S_2, and it is in excellent agreement with the measured curve S_3. Therefore, we rely on S_2 representing the real distribution of Sn(IV) in TiO_2. **Fig. S9D** visualizes the Sn-distribution in linear scale, clearly showing predominant surface localization of the dopant. We suggest that Sn(IV) is not fully integrated into the TiO_2 crystal structure but rather occupies metal vacancies at the TiO_2 surface.

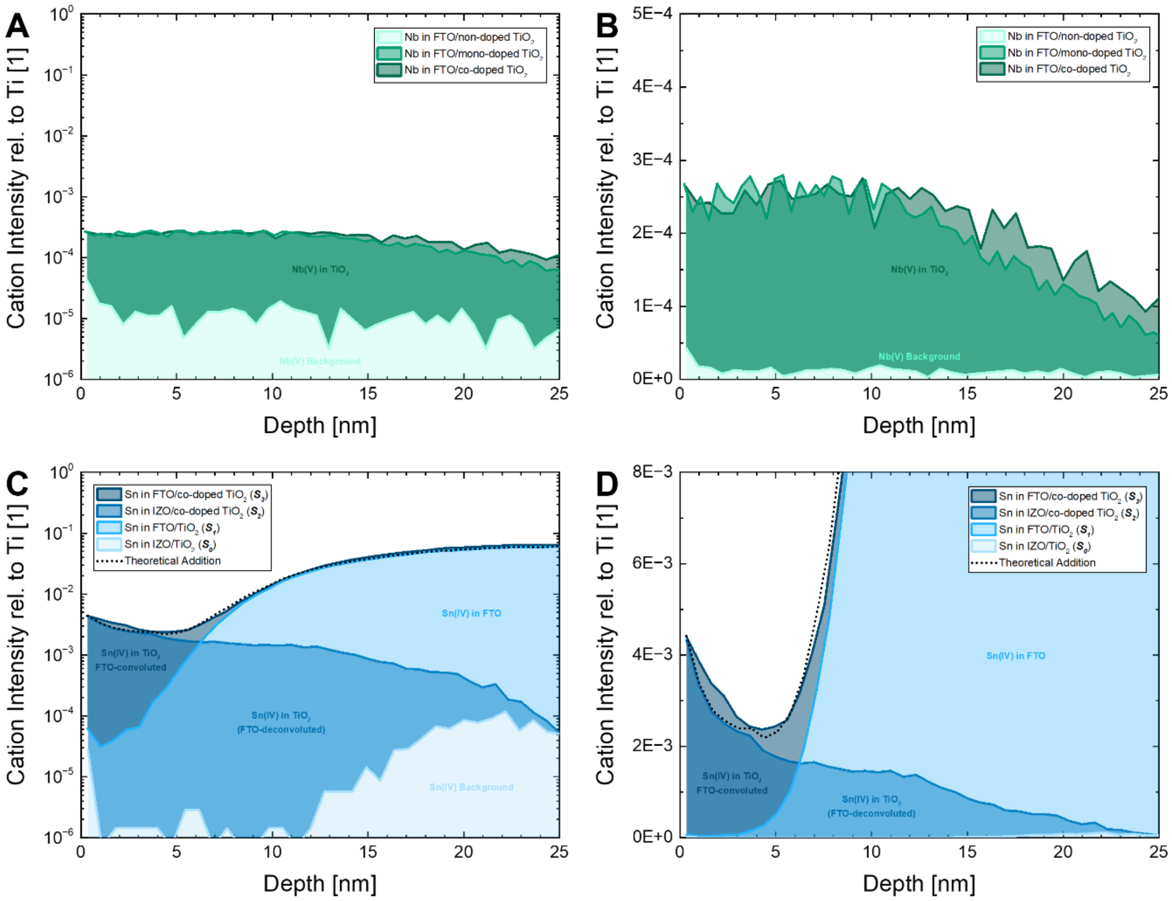

Fig. S9. **ToF-SIMS depth profiles reveal a homogeneous vertical distribution of Nb across the TiO_2 layer, while Sn is predominantly located at the TiO_2 surface. All curves were normalized to the maximum of the Ti-related signal.** The Nb-related signal of mono- and co-doped TiO_2 is reproducible and homogenous across TiO_2, as shown in **(A)** logarithmic and **(B)** linear scale. **(C)** Elucidation of the vertical distribution of Sn succeeded via separate measurement of non-doped TiO_2 on IZO (S_0), non-doped TiO_2 on FTO (S_1), co-doped TiO_2 on IZO (S_2), and co-doped TiO_2 on FTO (S_3). The numeric addition of the single components S_0, S_1, and S_2 (in dotted black) is in excellent agreement with the measured signal S_3. **(D)** In linear scale, the deconvoluted signal S_2 reveals Sn(IV) dopant to be predominantly located at the surface of TiO_2.

S3.4 HAXPES Measurements of (doped) TiO_2

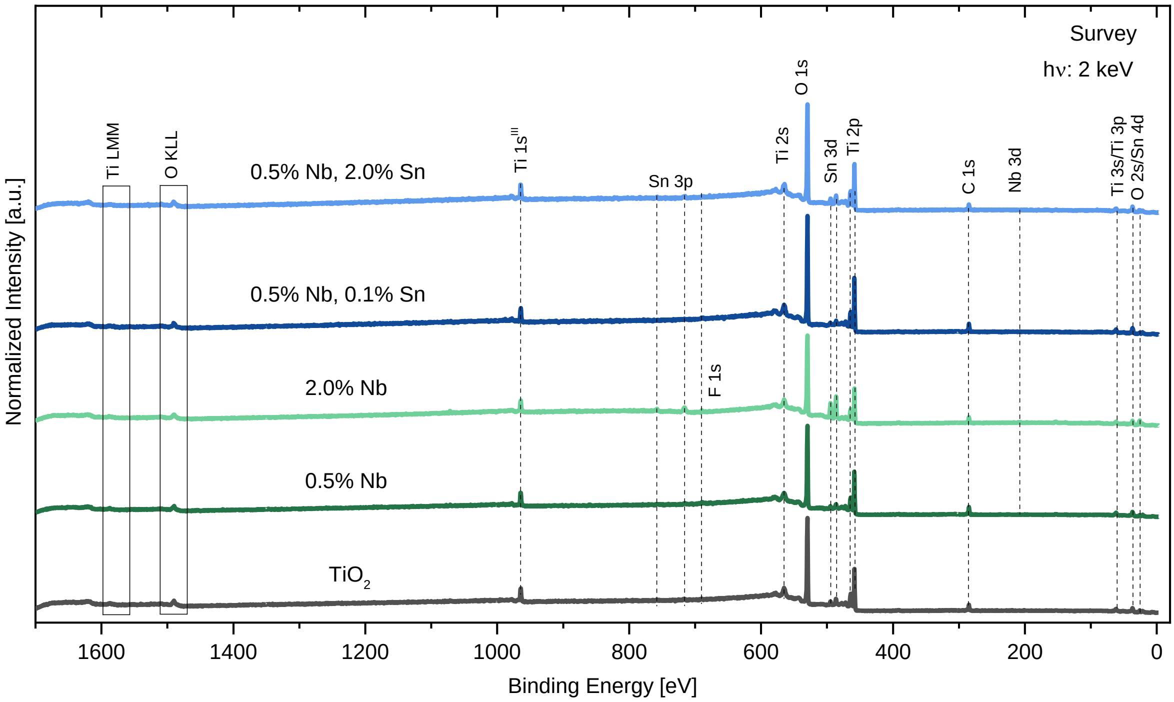

Fig. S10. HAXPES survey spectra of the investigated sample series. The spectra were measured using 2 keV excitation and normalized to background intensity, with vertical offsets added for clarity. Core level labels with superscripted numerals were excited by photons corresponding to higher diffraction orders of the monochromator (e.g., Ti $1s^{III}$ was excited by 6 keV photons from the third order of the monochromator settings).

Fig. S10 shows the survey spectra of the investigated sample series, which upon inspection reveal signal predominantly from Ti- and O-related lines (expected for the TiO_2 layers), and to a lesser extent from C-related lines, likely due to adventitious C from adsorbates. Because of the chosen low concentrations of (co-)dopants in the samples, Nb-related lines are not readily visible in the survey spectra. Surprisingly, Sn-related lines (e.g., the Sn 3d lines located within the BE range of 480 – 500 eV) are clearly seen for all measured samples (i.e., with and without Sn-doping), and in the case of samples prepared with Sn doping, the detected Sn-related signal is significantly larger than expected based on their nominal Sn concentration. These facts, in addition to the detection of F-related lines for all samples, point out that most of the measured Sn-related signal originates from the FTO substrate [also containing Sn(IV)]. As the probing depth of the 2 keV measurements is < 10 nm (see section of HAXPES experimental details above), which is significantly lower than the nominal thickness (i.e., 20 nm) of the TiO_2 layers, the detected “excess” Sn-related signal is likely due to pinholes and/or areas in the sample with TiO_2 thicknesses significantly thinner than the nominal value. For these reasons, we refrain from assessing the chemical environment and quantity of the Sn dopant in the sample series.

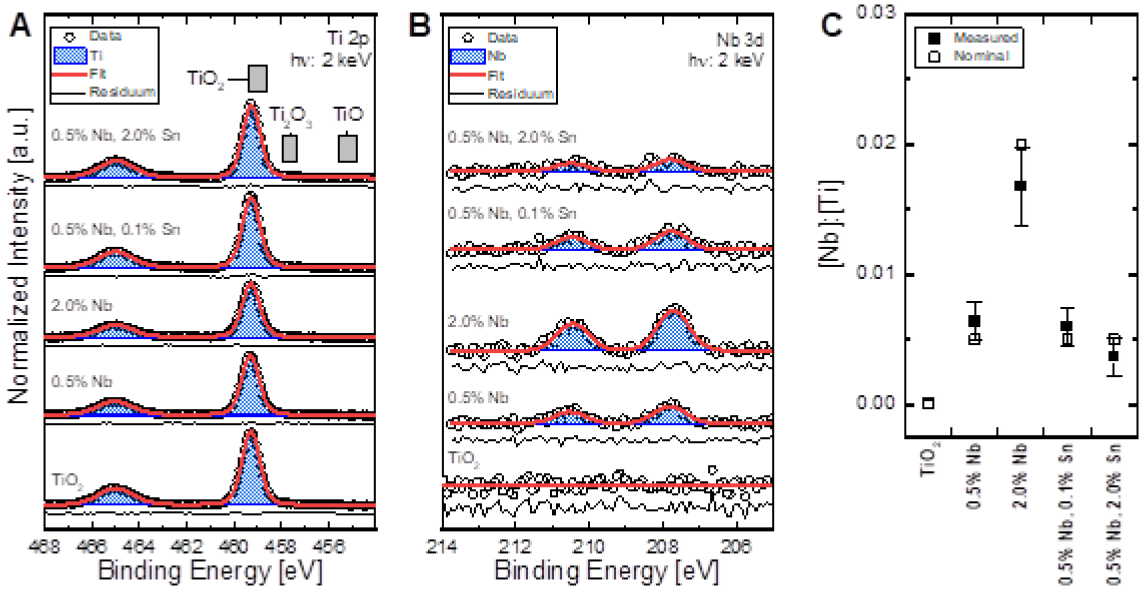

Fig. S11. **Nb(V) dopant levels agree with nominal values.** HAXPES detail spectra of the **(A)** Ti 2p and **(B)** Nb 3d energy regions of the differently (co-)doped TiO_2 samples. The spectra were measured using a 2 keV excitation and normalized to background intensity, with vertical offsets added for clarity. Curve fit results are included. The gray-filled areas in (A) are binding energy (BE) ranges reported in the literature for Ti-based reference compounds.[12] **(C)** [Nb]:[Ti] surface composition of variously treated TiO_2 films, determined from the HAXPES measurements shown in (A) and (B).

S3.5 SEM Measurements of $TiO_2/CsPbI_3$ Heterojunctions

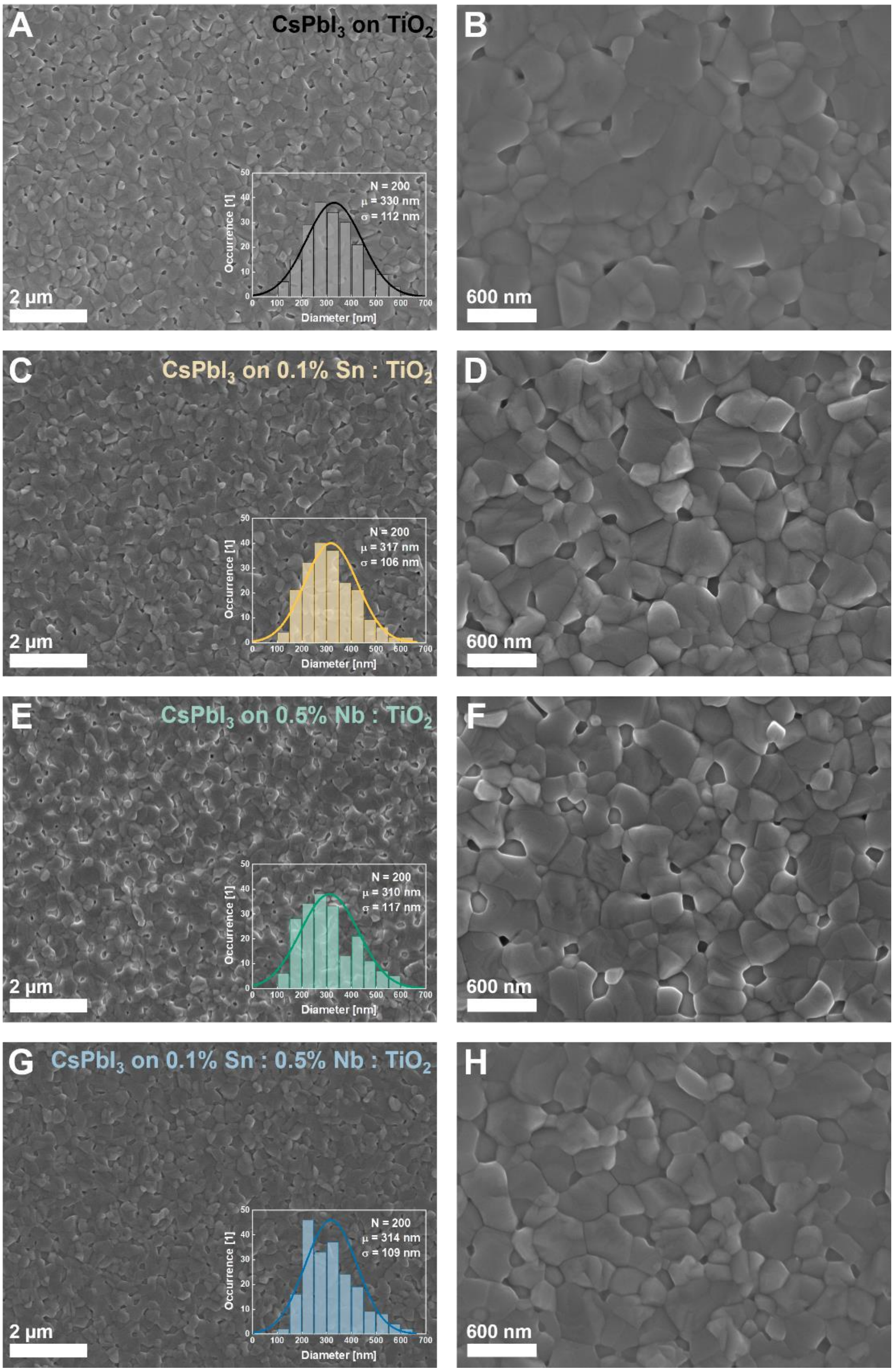

Fig. S12. The grain morphology of $CsPbI_3$ is not affected by the type of doping in TiO_2 substrate. SEM micrographs at 10k and 30k magnification, respectively, of $CsPbI_3$ films deposited on **(A, B)** non-doped TiO_2, **(C, D)** 0.1% Sn(IV) mono-doped TiO_2, **(E, F)** 0.5% Nb(V) mono-doped TiO_2, and **(G, H)** 0.1% Sn(IV) and 0.5% Nb(V) co-doped TiO_2.

S3.6 XRD Measurements of $TiO_2/CsPbI_3$ Heterojunctions

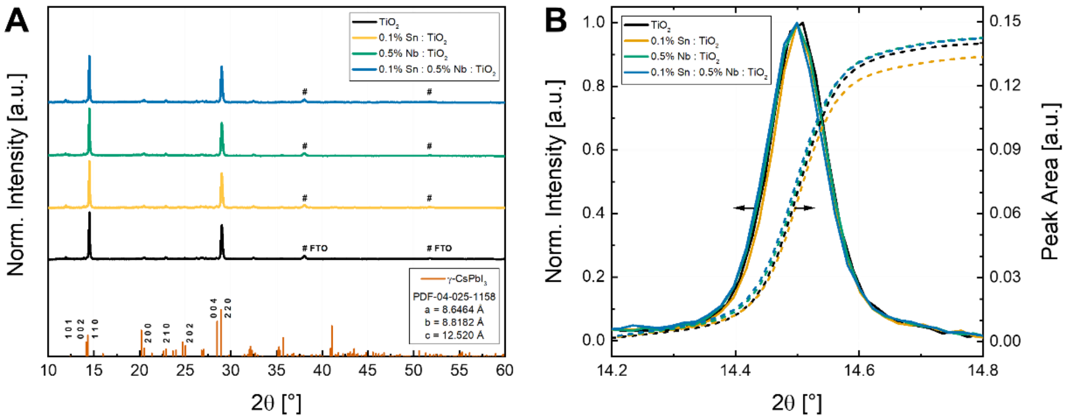

Fig. S13. **The crystallinity of $CsPbI_3$ deposited on TiO_2 substrates is not affected by the doping level. (A)** The X-ray diffractograms of $CsPbI_3$ deposited on non-, Sn(IV) mono-, Nb(V) mono-, and Sn(IV) and Nb(V) co-doped TiO_2 show no differences. **(B)** Integration of the 1 1 0 reflection at 14.5° 2θ results in an equal peak area. The absence of line narrowing or broadening confirms equal crystallite size distributions.

S3.7 ssPL Measurements of $TiO_2/CsPbI_3$ Heterojunctions

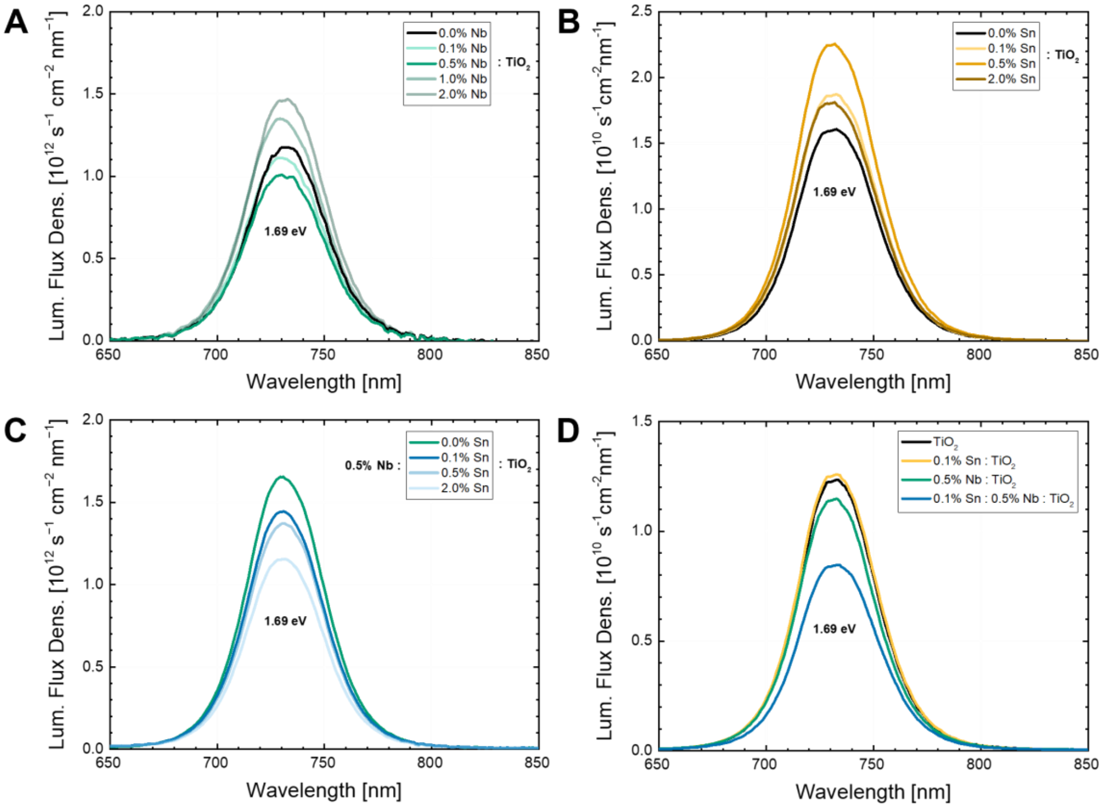

Fig. S14. **(A)** A concentration series of Nb(V) mono-doped in TiO_2, the $CsPbI_3$ PL intensity experiences maximum quenching at 0.5 mol% Nb(V). **(B)** In contrast, the PL intensity of $CsPbI_3$ is not quenched for any concentration of Sn(IV) mono-doped in TiO_2. **(C)** Introducing Sn(IV) co-dopant into Nb(V) mono-doped TiO_2 leads to ssPL quenching proportional to the Sn(IV) doping level. **(D)** Direct comparison of $CsPbI_3$ PL spectra on non-, mono-, and co-doped TiO_2. While Sn(IV) mono-doping slightly enhances PL intensity, Nb(V) mono-doping and Sn(IV) and Nb(V) co-doping lead to PL quenching.

S3.8 trPL Measurements of TiO_2/$CsPbI_3$ Heterojunctions

The PL transient curve was fitted by an arbitrary sum of exponential slopes to compute the differential lifetime. The differential lifetime was calculated as described in the literature.[13]

(Eq. S3)
$$\tau_{diff} = -\left[\frac{2d\left(ln\left(trPL(t)\right)\right)}{dt}\right]^{-1}$$

For differential lifetimes plots, the QFLS was estimated via **Eq. S4** and **Eq. S5**:

(Eq. S4)
$$PL = k_{rad} \cdot n \cdot p = k_{rad} \cdot n_i^2 \text{ (for intrinsic SCs)}$$

(Eq. S5)
$$QFLS = kT \cdot ln\left(\frac{PL}{PL(0)}\right) + kT \cdot ln\left(\frac{n(0)^2}{n_i^2}\right) \text{ with } n_i^2 = N_c \cdot N_v \cdot e^{\frac{-E_g}{kT}}$$

With the assumption of $N_c = N_v = 2 \cdot 10^{18}$ cm^{-3}. This value determines the starting QFLS(0). The other values are determined by the normalized PL decay PL/PL(0), respectively.

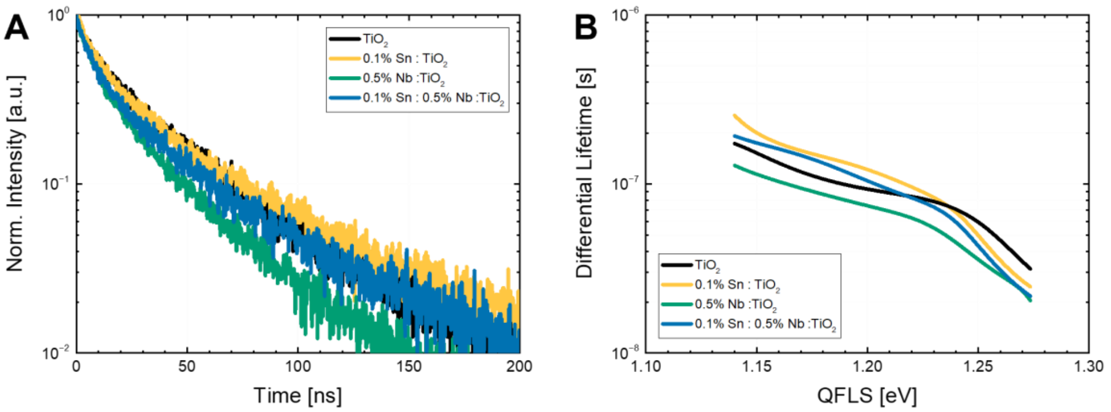

Fig. S15. Non-radiative recombination is reduced in $CsPbI_3$ on co-doped TiO_2. (A) trPL decay for a series of $CsPbI_3$ on non-, Sn(IV) mono-, Nb(V) mono-, and Sn(IV) and Nb(V) co-doped TiO_2. **(B)** Differential lifetime analysis was achieved via 6-fold exponential fitting of the PL transients in (A). $CsPbI_3$ on Nb(V) mono- and co-doped TiO_2 shows the fastest differential lifetime at high QFLS, indicating fast electron extraction, while differential lifetimes for $CsPbI_3$ on co-doped TiO_2 is consistently higher than for $CsPbI_3$ on Nb(V) mono-doped TiO_2 at lower QFLS, showing reduced non-radiative recombination.

S3.9 trSPV Measurements of TiO_2/$CsPbI_3$ Heterojunctions

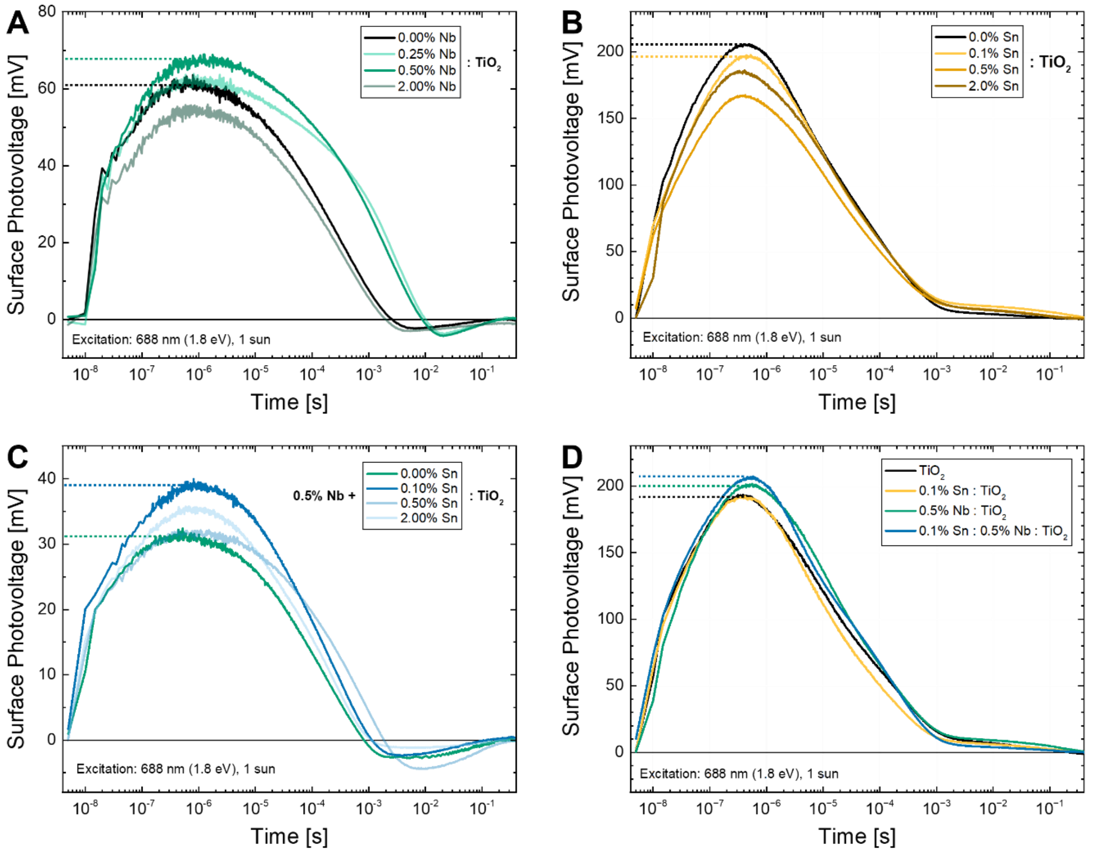

Fig. S16. **TiO_2 co-doped with 0.1% Sn(IV) and 0.5% Nb(V) consistently shows improved electron extraction from $CsPbI_3$, as shown via trSPV. (A)** 0.5% Nb(V) exhibits highest absolute amplitude and slowest charge recombination in a series of increasing mono-dopant concentrations. **(B)** In contrast, none of the tested Sn(IV) mono-dopant concentrations improves electron extraction with respect to the non-doped control. **(C)** Screening different Sn(IV) concentrations while keeping the Nb(V) at constant 0.5 mol% reveals improved electron extraction when Nb(V) is combined with 0.1 mol% Sn(IV) co-dopant. **(D)** Control experiment confirming improved electron extraction of co-doped TiO_2 with respect to both mono- and non-doped TiO_2.

S3.10 *J-V* Statistics

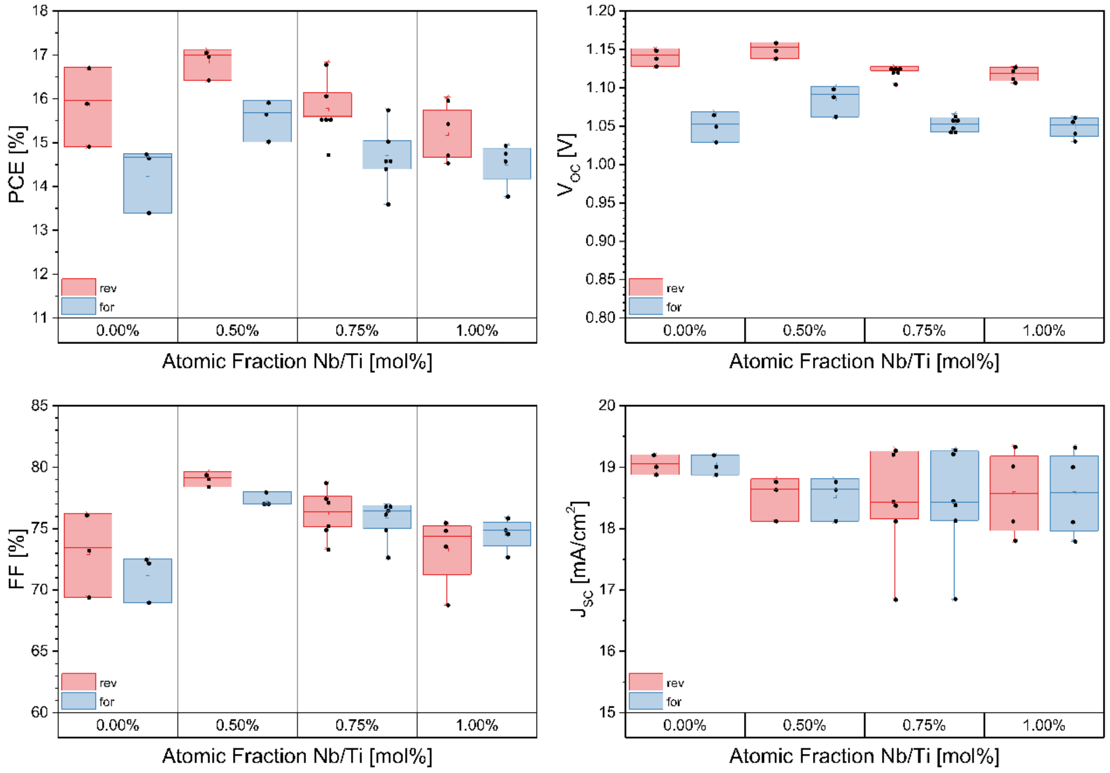

Fig. S17. Figures of merit for $CsPbI_3$-based solar cells with different doping concentrations of Nb(V) with respect to TiO_2.

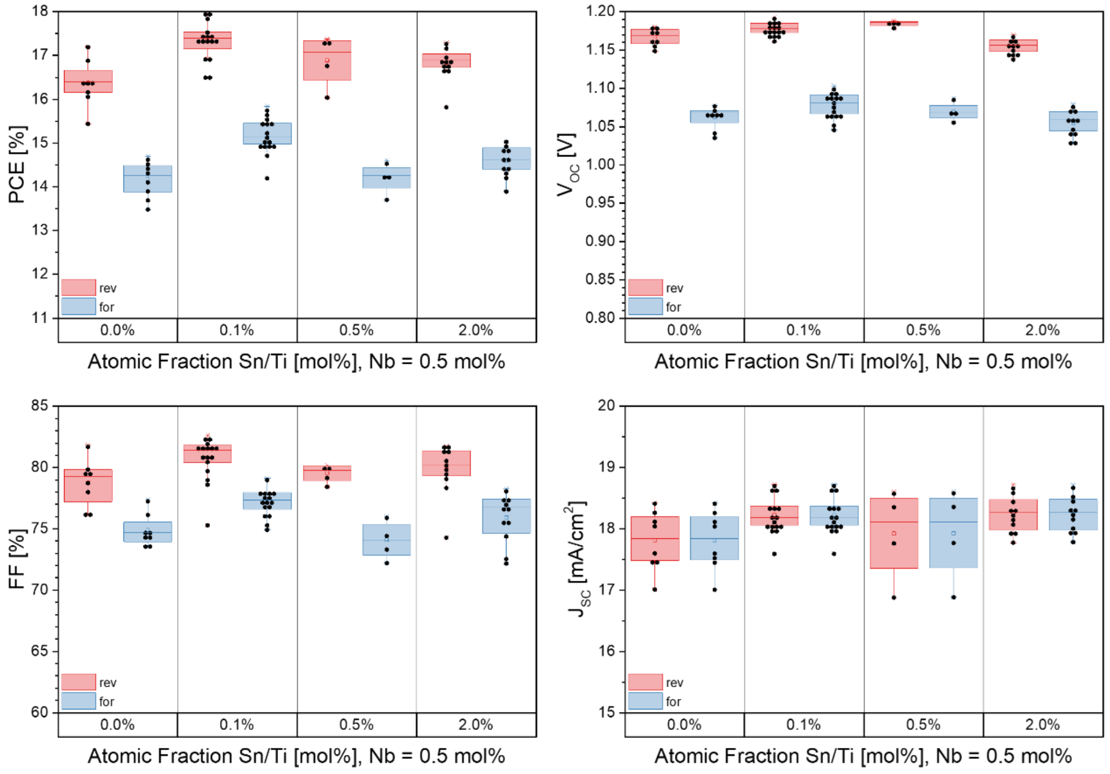

Fig. S18. Figures of merit for $CsPbI_3$-based solar cells with different doping concentrations of Sn(IV) with respect to TiO_2. The concentration of Nb(V) was kept constant at 0.5 mol%.

S3.11 EQE Measurements

EQE spectra of the perovskite solar cells were obtained after referencing the measured current I_{cell} to a silicon solar cell according to **Eq. S6**, where h represents the Planck constant, c represents the speed of light, q represents the elementary charge, λ represents the wavelength, I_{ref} represents the current of the reference cell, and R_{ref} representing the responsivity parameter of the reference detector.

(Eq. S6)
$$EQE(\lambda) = 100\% \cdot \frac{hc}{q\lambda} \cdot \frac{I_{cell}}{I_{ref}R_{ref}}$$

The short circuit current density (J_{SC}) was obtained via integration of the measured EQE spectrum and the reference solar spectral irradiance at air mass 1.5 global ($\Phi_{AM1.5G}$) in the wavelength range between 300 nm and 800 nm, according to **Eq. S7**.

(Eq. S7)
$$J_{SC} = \int_{\lambda_{min}}^{\lambda_{max}} \Phi_{AM1.5G}(\lambda) \cdot EQE(\lambda)\, d\lambda$$

The radiative dark saturation current density (J_0^{rad}) was obtained via **Eq. S8** where Φ_{BB} is the black body spectral radiance. Φ_{BB} was calculated for a temperature (T) of 293.15 K using Planck's law according to **Eq. S9**, with k_B representing the Boltzmann constant.

(Eq. S8)
$$J_0^{rad} = q \cdot \int_0^{\infty} \Phi_{BB}^{293K} \cdot EQE(E)\, dE$$

(Eq. S9)
$$\Phi_{\mathrm{BB}} = \frac{2\pi}{h^3c^2} \cdot \frac{E^2}{\exp\left(\frac{E}{k_BT}\right) - 1}$$

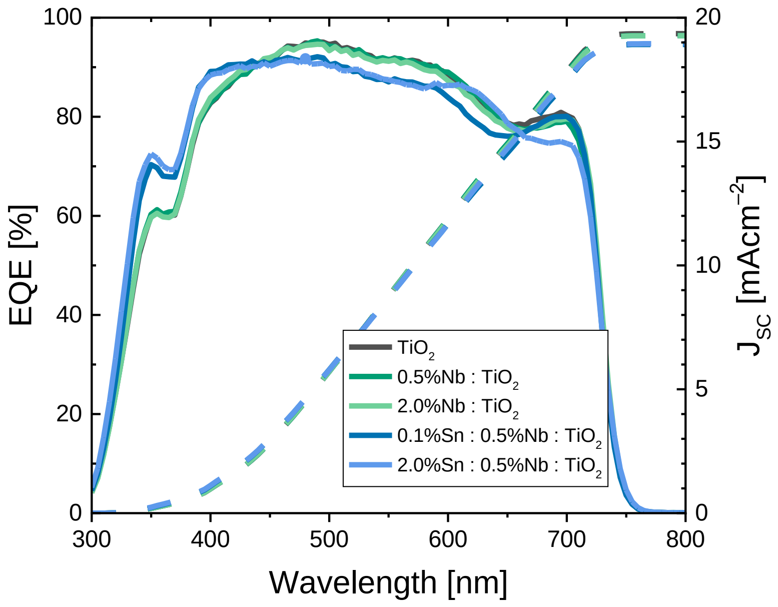

Fig. S19. EQE spectra of full solar cells with varying Nb(V) and Sn(IV) dopant concentrations in TiO_2.

Tab. S3. Dark thermal recombination current J_0^{rad} and J_{SC} extracted from EQE spectra.

Composition Host Material	Nb(V)/Ti(IV) [mol%]	Sn(IV)/Ti(IV) [mol%]	J_0^{rad} [10^{-21} A m^{-2}]	J_{SC} [mA cm^{-2}]
TiO_2	0	0	2.72	19.4
TiO_2	0.5	0	2.55	19.3
TiO_2	2.0	0	2.62	19.3
TiO_2	0.5	0.1	2.75	18.9
TiO_2	0.5	2.0	2.75	19.0

S3.12 Long-Term Maximum Power Point Tracking

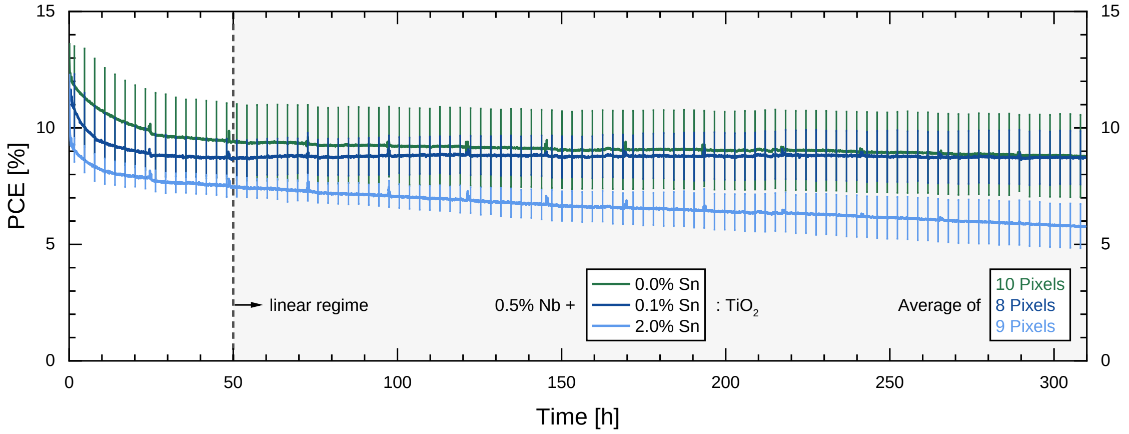

Fig. S20. Absolute device performance tested in MPP-tracking under continuous AM1.5G illumination, according to ISOS-L1-I protocol. The discrepancy between the PCE implied in *J*-*V*-statistics (**Fig. S18**) and the shown LT-MPP experiment arises from the use of a UV-filter. The UV-filter was justified to minimize photocatalytic activity of TiO_2[14] in order to single out the contribution of $CsPbI_3$ on the solar cell degradation. The co-doped champion does not exhibit significant performance loss over 300 h after the initial burn-in phase.

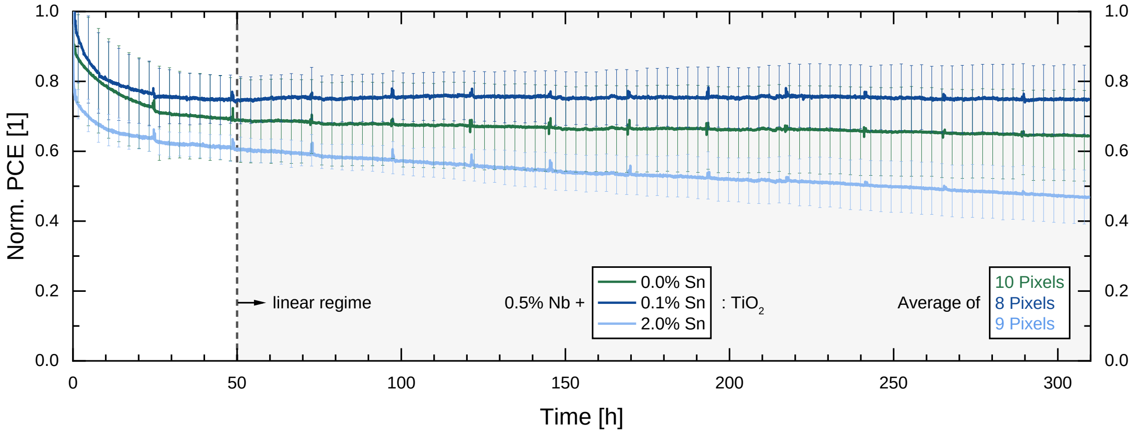

Fig. S21. Normalized device performance tested in MPP-tracking (s. **Fig. S20**) under continuous AM1.5G illumination. The co-doped champion does not exhibit significant performance loss over 300 h after the initial burn-in phase.

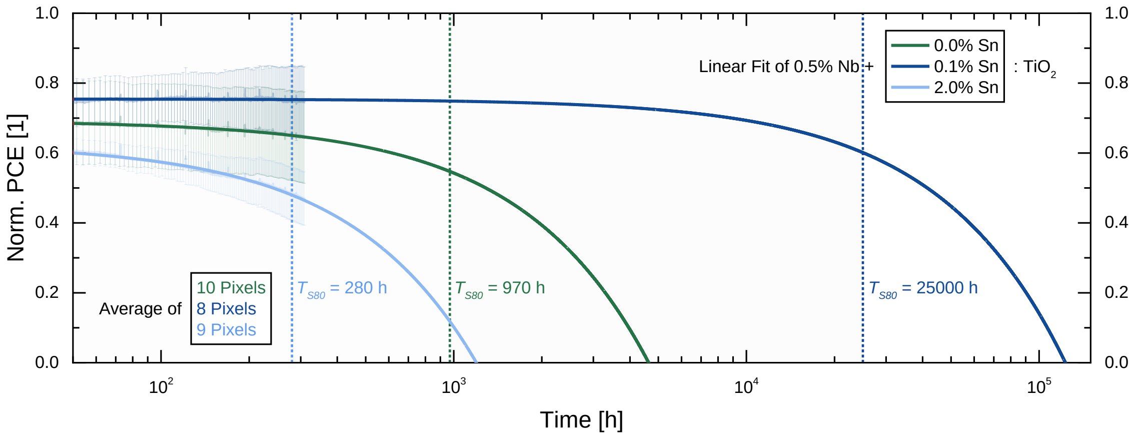

Fig. S22. Linear fit of the normalized MPP-tracking shown in **Fig. S21** in a single-logarithmic plot. The determined slopes (s. **Tab. S4**) lead to projected T_{S80} lifetimes of 970 h, 25'000 h and 280 h for $CsPbI_3$ solar cells including the mono-doped, co-doped and over-doped TiO_2, respectively.

Tab. S4. Overview of different Sn(IV) doping levels and associated stability decay extracted from linear fit of the region after the burn-in phase at 150 h.

Atomic Fraction Sn/Ti [%]	Slope in Linear Regime [10^{-4} % h^{-1}]	Projected T_{S80} [h]
0.00	(–1.50 ± 0.01)	280
0.10	(–0.06 ± 0.01)	970
2.00	(–5.24 ± 0.01)	25000

S3.13 Drift-Diffusion Simulations

The transient SPV data presented in the main text was fitted by drift-diffusion (DD) simulations. We used the Silvaco Atlas commercial software to numerically solve the Poisson equation, together with the carrier continuity equations in a 2D mesh of points, which we assigned either to the perovskite ($CsPbI_3$), or to TiO_2. These two regions were both shaped as rectangles and placed one on top of the other. Zero-current boundary conditions were assigned to the top $CsPbI_3$ surface, as well as the lateral surfaces. At the back TiO_2 surface a Schottky-type contact with variable WF was placed to mimic the presence of the highly conductive FTO substrate.

The set of DD equations is reported below:

(Eq. S10)
$$\frac{\partial E}{\partial x} = \frac{q}{\varepsilon}[p(x) - n(x) - N_A^- + N_D^+]$$

(Eq. S11)
$$\frac{\partial n}{\partial t} = \frac{1}{q}\frac{\partial J_n}{\partial x} + G_n - R_n$$

(Eq. S12)
$$\frac{\partial p}{\partial t} = -\frac{1}{q}\frac{\partial J_p}{\partial x} + G_p - R_p$$

(Eq. S13)
$$J_n = q\mu_n nE + qD_n\frac{\partial n}{\partial x}$$

(Eq. S14)
$$SPV(t) = \varphi(x = 0, t) - \varphi(x = 0, t = 0)$$

The SPV is calculated from the electrostatic potential φ at $x = 0$ (the perovskite surface), with respect to φ at the beginning of the simulation time ($t = 0$), i.e. under dark. The ($G_n - R_n$) term can be expressed as:

(Eq. S15)
$$G_n - R_n = G_{OPT} - (R_{b,SRH} + R_{An} + C^{OPT}(np - n_{ie}^2) + \mathrm{C}^{\mathrm{AUG,n}}(pn^2 - nn_{ie}^2) + \mathrm{C}^{\mathrm{AUG,p}}(np^2 - pn_{ie}^2) + R_{interf} + R_{Dn})$$

Where G_{OPT} refers to optical generation following the Beer-Lambert law, $R_{b,SRH}$ to bulk non-radiative recombination with the Shockley-Read-Hall (SRH) formalism, R_{An} and R_{Dn} are the net capture-emission rates for electrons via shallow acceptor traps in the perovskite, and deep donor traps in TiO_2, respectively, the fourth term refers to bulk radiative recombination with the parameter C^{OPT}, the fifth and sixth terms refer to Auger recombination with the two parameters $C^{AUG,n}$ and $C^{AUG,p}$, and R_{interf} represents non-radiative recombination at the $CsPbI_3/TiO_2$ interface.

To reproduce the transient SPV measurement, we ran the Atlas solver in time-dependent mode, obtaining the solution of the DD equations for each point in time, spanning about 10 orders of magnitude. At the beginning of the simulation time, a 5 ns laser pulse with constant intensity (from $t = 0$ to $t = 5$ ns) was applied on the perovskite surface, leaving the structure under dark for the rest of the simulation.

The optoelectronic parameters assigned to $CsPbI_3$ and to TiO_2 are reported in **Tab. S5** to **Tab. S8**. **Tab. S5** and **Tab. S6** report the fitting parameters for the mono-doped TiO_2 and the co-doped TiO_2 transient SPV curves (respectively) with the initial parameter set, with the bulk perovskite possessing neutral mid-gap non-radiative recombination centers. **Tab. S7** and **Tab. S8** report the fitting parameters for the same curves with the addition of bulk shallow acceptor traps in the $CsPbI_3$ layer.

Parameters with # were variable and were employed to fit the transient SPV curves. For this purpose, we employed an in-house developed code, together with the MatLab functions "ga" and "lsqcurvefit", to feed the DD model the fitting parameters and consequently obtain a best-fit curve of the experimental data.

We chose certain parameters to be independent of the doping nature of TiO_2 (indicated in yellow boxes in **Tab. S5** to **Tab. S8**), such as the laser fluence F_{laser}, the $CsPbI_3$ electron affinity χ_{pero}, the back contact WF, the carrier mobility in both layers ($\mu_e = \mu_h$, considered equal for electrons and holes), the non-radiative characteristic time in $CsPbI_3$ ($\tau_e = \tau_h$, considered equal for electrons and holes), and the $CsPbI_3$ shallow acceptor trap parameters, such as the volume density N_{tA}, the energy level $E_{tA} - E_{CBM}$, and the charge carrier capture cross sections for electrons and holes (σ_{etA}, and σ_{htA}, respectively). Other parameters, such as the non-radiative recombination velocity at the $CsPbI_3/TiO_2$ interface ($v_{interf,e}$ and $v_{interf,h}$), and the donor trap parameters in TiO_2 (N_{tD}, $E_{tD} - E_{VBM}$, σ_{etD} and σ_{htD}), were fitted independently on each curve.

To allow for non-radiative recombination of carriers at the $CsPbI_3/TiO_2$ interface, we included a 1 nm thick recombination layer ($d_{interf} = 1$ nm) close to the interface where non-radiative Shockley-Read-Hall recombination is activated, and the hole and electron characteristic times are specified ($\tau_{interf,e} = \tau_{interf,h}$, respectively). We have converted these values into carrier non-radiative recombination velocities ($v_{interf,e}$ and $v_{interf,h}$), according to $v_{interf,} = d_{interf}\, \tau_{interf}^{-1}$.

S3.13.1 Parameter Tables

Tab. S5. Parameter table for the $CsPbI_3$/mono-doped TiO_2 fit without acceptor traps in the $CsPbI_3$ layer. Parameters with # are the result of the fit. The best-fit value for the contact WF was 4.42 eV. Boxes in yellow contain fitting parameters shared between **Tab. S5** and **Tab. S6**. Parameters with asterisk are based on measurements by the authors.

Parameter	Symbol	Unit	$CsPbI_3$	Interface	TiO_2
Laser Fluence	F_{laser}	$J\ cm^{-2}$		1.581×10^{-8}#	
Thickness	h	nm	350*	1	20*
Bandgap	E_g	eV	1.72[15]	1.72[15]	3.335*
Electron Affinity	χ	eV	3.373#	3.373#	3.53*
Rel. Permittivity	ϵ		30	30	31[16]
Effective Cond. Band DOS	N_C	cm^{-3}	10^{19}	10^{19}	10^{19}
Effective Val. Band DOS	N_V	cm^{-3}	10^{19}	10^{19}	10^{19}
Mobility, Electrons and Holes	$\mu_e = \mu_h$	$cm^2\ V^{-1} s^{-1}$	1.417#	1.417#	7.131×10^{-4}#
Lifetime, Electrons	τ_e	µs	0.721#	1.205×10^{-15} s $\rightarrow v_{interf,e} = 8.299 \times 10^{7}$ cm s^{-1}#	/
Lifetime, Holes	τ_h	µs	0.721#	3.510×10^{-9} s $\rightarrow v_{interf,h} = 2.849 \times 10^{1}$ cm s^{-1}#	/
Trap Density (Acceptor, Donor)	N_A, N_D	cm^{-3}	$N_{tA} = 0$#	/	$N_{tD} = 1.069 \times 10^{20}$#
Trap Energy	E_t	eV	/	/	$E_{tD} - E_{VBM} = 2.688$#
Trap Electron Capture Cross Section	σ_e	cm^2	/	/	$\sigma_{etD} = 7.251 \times 10^{-13}$#
Trap Hole Capture Cross Section	σ_h	cm^2	/	/	$\sigma_{htD} = 10^{-14}$

Tab. S6. Parameter table for the $CsPbI_3$/co-doped TiO_2 fit without acceptor traps in the $CsPbI_3$ layer. Parameters with # are the result of the fit. The best-fit value for the contact WF was 4.42 eV. Boxes in yellow contain fitting parameters shared between **Tab. S5** and **Tab. S6**. Parameters with asterisk are based on measurements by the authors.

Parameter	Symbol	Unit	$CsPbI_3$	Interface	TiO_2
Laser Fluence	F_{laser}	J cm^2		1.581×10^{-8}#	
Thickness	h	nm	350*	1	20*
Bandgap	E_g	eV	1.72[15]	1.72[15]	3.335*
Electron Affinity	χ	eV	3.373#	3.373#	3.53*
Rel. Permittivity	ϵ		30	30	31[16]
Effective Cond. Band DOS	N_C	cm^{-3}	10^{19}	10^{19}	10^{19}
Effective Val. Band DOS	N_V	cm^{-3}	10^{19}	10^{19}	10^{19}
Mobility, Electrons and Holes	$\mu_e = \mu_h$	$cm^2\ V^{-1}s^{-1}$	1.417#	1.417#	7.131×10^{-4}#
Lifetime, Electrons	τ_e	µs	0.721#	1.056×10^{-15} s $\rightarrow v_{interf,e} = 9.465 \times 10^{7}$ cm s^{-1}#	/
Lifetime, Holes	τ_h	µs	0.721#	2.530×10^{-6} s $\rightarrow v_{interf,h} = 3.953 \times 10^{-2}$ cm s^{-1}#	/
Trap Density (Acceptor, Donor)	N_A, N_D	cm^{-3}	$N_{tA} = 0$	/	$N_{tD} = 9.629 \times 10^{19}$#
Trap Energy	E_t	eV	/	/	$E_{tD} - E_{VBM} = 2.815$#
Trap Electron Capture Cross Section	σ_e	cm^2	/	/	$\sigma_{etD} = 2.440 \times 10^{-13}$#
Trap Hole Capture Cross Section	σ_h	cm^2	/	/	$\sigma_{htD} = 10^{-14}$

Tab. S7. Parameter table for the $CsPbI_3$/mono-doped TiO_2 fit with acceptor traps in the $CsPbI_3$ layer. Parameters with # are the result of the fit. The best-fit value for the contact WF was 4.35 eV. Boxes in yellow contain fitting parameters shared between **Tab. S7** and **Tab. S8**. Parameters with asterisk are based on measurements by the authors.

Parameter	Symbol	Unit	$CsPbI_3$	Interface	TiO_2
Laser Fluence	F_{laser}	J cm^2		1.758×10^{-8}#	
Thickness	h	nm	350*	1	20*
Bandgap	E_g	eV	1.72[15]	1.72[15]	3.335*
Electron Affinity	χ	eV	3.352#	3.352#	3.53*
Rel. Permittivity	ϵ		30	30	31[16]
Effective Cond. Band DOS	N_C	cm^{-3}	10^{19}	10^{19}	10^{19}
Effective Val. Band DOS	N_V	cm^{-3}	10^{19}	10^{19}	10^{19}
Mobility, Electrons and Holes	$\mu_e = \mu_h$	$cm^2\ V^{-1}s^{-1}$	3.951#	3.951#	1.077×10^{-3}#
Lifetime, Electrons	τ_e	µs	1.138#	1.535×10^{-15} s $\rightarrow v_{interf,e} = 6.513 \times 10^{7}$ cm s^{-1}#	/
Lifetime, Holes	τ_h	µs	1.138#	5.877×10^{-9} s $\rightarrow v_{interf,h} = 1.702 \times 10^{1}$ cm s^{-1}#	/
Trap Density (Acceptor, Donor)	N_A, N_D	cm^{-3}	$N_{tA} = 6.352 \times 10^{18}$#	/	$N_{tD} = 7.658 \times 10^{19}$#
Trap Energy	E_t	eV	$E_{tA} - E_{CBM} = -0.045$#	/	$E_{tD} - E_{VBM} = 2.673$#
Trap Electron Capture Cross Section	σ_e	cm^2	$\sigma_{etA} = 1.557 \times 10^{-18}$#	/	$\sigma_{etD} = 4.887 \times 10^{-13}$#
Trap Hole Capture Cross Section	σ_h	cm^2	$\sigma_{htA} = 3.770 \times 10^{-16}$#	/	$\sigma_{htD} = 10^{-14}$

Tab. S8. Parameter table for the $CsPbI_3$/co-doped TiO_2 fit with acceptor traps in the $CsPbI_3$ layer. Parameters with # are the result of the fit. The best-fit value for the contact WF was 4.35 eV. Boxes in yellow contain fitting parameters shared between **Tab. S7** and **Tab. S8**. Parameters with asterisk are based on measurements by the authors.

Parameter	Symbol	Unit	$CsPbI_3$	Interface	TiO_2
Laser Fluence	F_{laser}	J cm^2		1.758×10^{-8}#	
Thickness	h	nm	350*	1	20*
Bandgap	E_g	eV	1.72[15]	1.72[15]	3.335*
Electron Affinity	χ	eV	3.352#	3.352#	3.53*
Rel. Permittivity	ϵ		30	30	31[16]
Effective Cond. Band DOS	N_C	cm^{-3}	10^{19}	10^{19}	10^{19}
Effective Val. Band DOS	N_V	cm^{-3}	10^{19}	10^{19}	10^{19}
Mobility, Electrons and Holes	$\mu_e = \mu_h$	$cm^2\ V^{-1}s^{-1}$	3.951#	3.951#	$1.077\ 10^{-3}$#
Lifetime, Electrons	τ_e	µs	1.138#	1.52×10^{-15} s $\rightarrow v_{interf,e} = 6.575\times10^{7}$ cm s^{-1}#	/
Lifetime, Holes	τ_h	µs	1.138#	1.03×10^{-6} s $\rightarrow v_{interf,h} = 9.683\times10^{-2}$ cm s^{-1}#	/
Trap Density (Acceptor, Donor)	N_A, N_D	cm^{-3}	$N_{tA} = 6.352\times10^{18}$#	/	$N_{tD} = 7.521\times10^{19}$#
Trap Energy	E_t	eV	$E_{tA} - E_{CBM} = -0.045$#	/	$E_{tD} - E_{VBM} = 2.758$#
Trap Electron Capture Cross Section	σ_e	cm^2	$\sigma_{etA} = 1.557\times10^{-18}$#	/	$\sigma_{etD} = 4.732\times10^{-13}$#
Trap Hole Capture Cross Section	σ_h	cm^2	$\sigma_{htA} = 3.770\times10^{-16}$#	/	$\sigma_{htD} = 10^{-14}$

S3.13.2 Best-Fit trSPV Curves

Below, we report the best-fit trSPV curves and band diagrams obtained with the parameters listed in **Tab. S5** to **Tab. S8**.

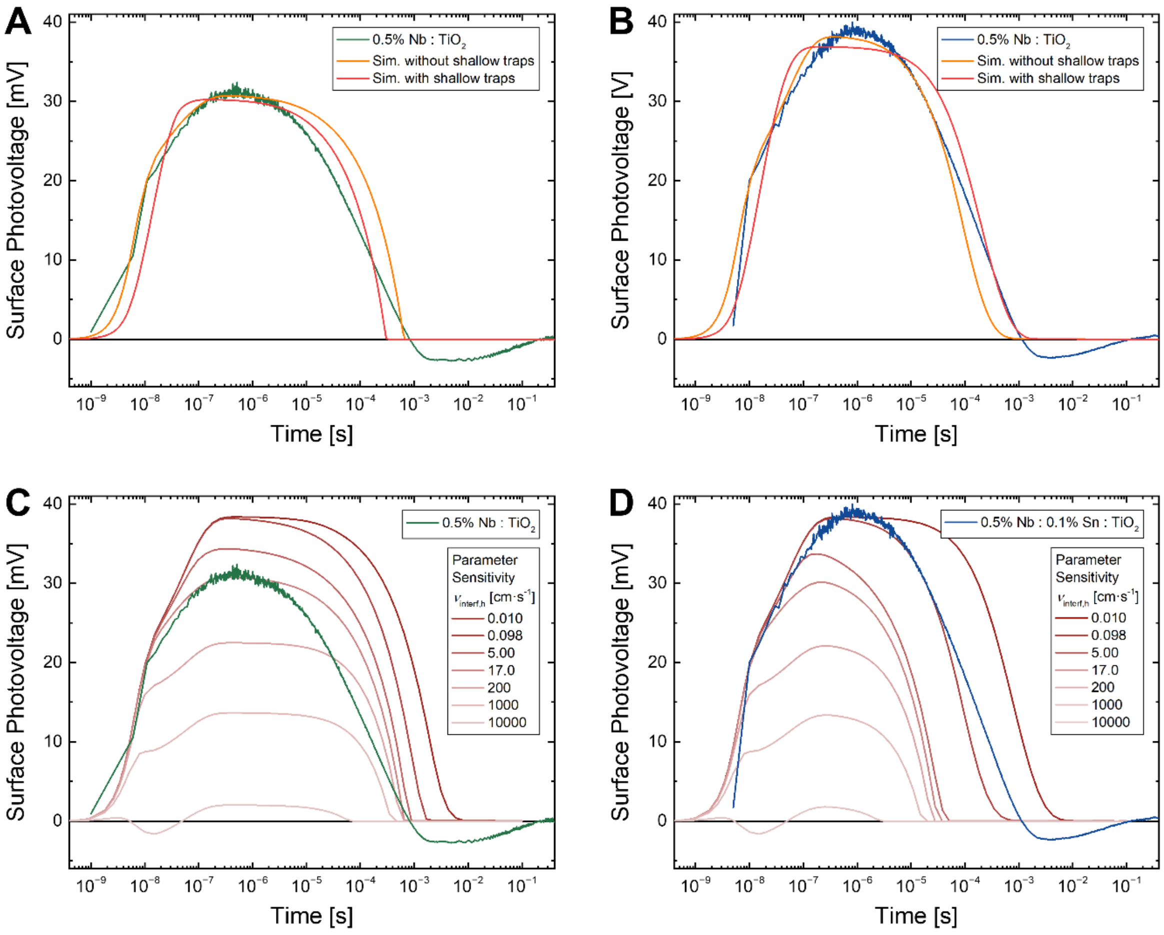

Fig. S23. **(A, B)** Effect of the consideration of shallow traps in $CsPbI_3$ in the DD simulation (red vs. orange curves). Best-fit trSPV curves were obtained with the parameters listed in **Tab. S5** to **Tab. S8**. Fit of (A) mono-doped and (B) and co-doped TiO_2 without (orange) and with (red) bulk shallow traps in $CsPbI_3$. **(C, D)** In both curves, the signal height is sensitive to the interface hole recombination velocity $v_{interf,h}$. Fitting the original data yields (C) 17.0 cms^{-1} for mono-doped TiO_2 and (D) 0.098 cms^{-1} for co-doped TiO_2.

In mono- or co-doped TiO_2 we notice a potential drop (**s. Fig. S24**), which is a result of the mismatch between the contact WF (ϕ_{cont} = 4.35 eV) and the TiO_2 WF at the top interface (ϕ_{co} = 4.003 eV, ϕ_{mono} = 4.085 eV). The large potential drop in TiO_2 is due to the large donor trap concentration, partially screening the back contact.

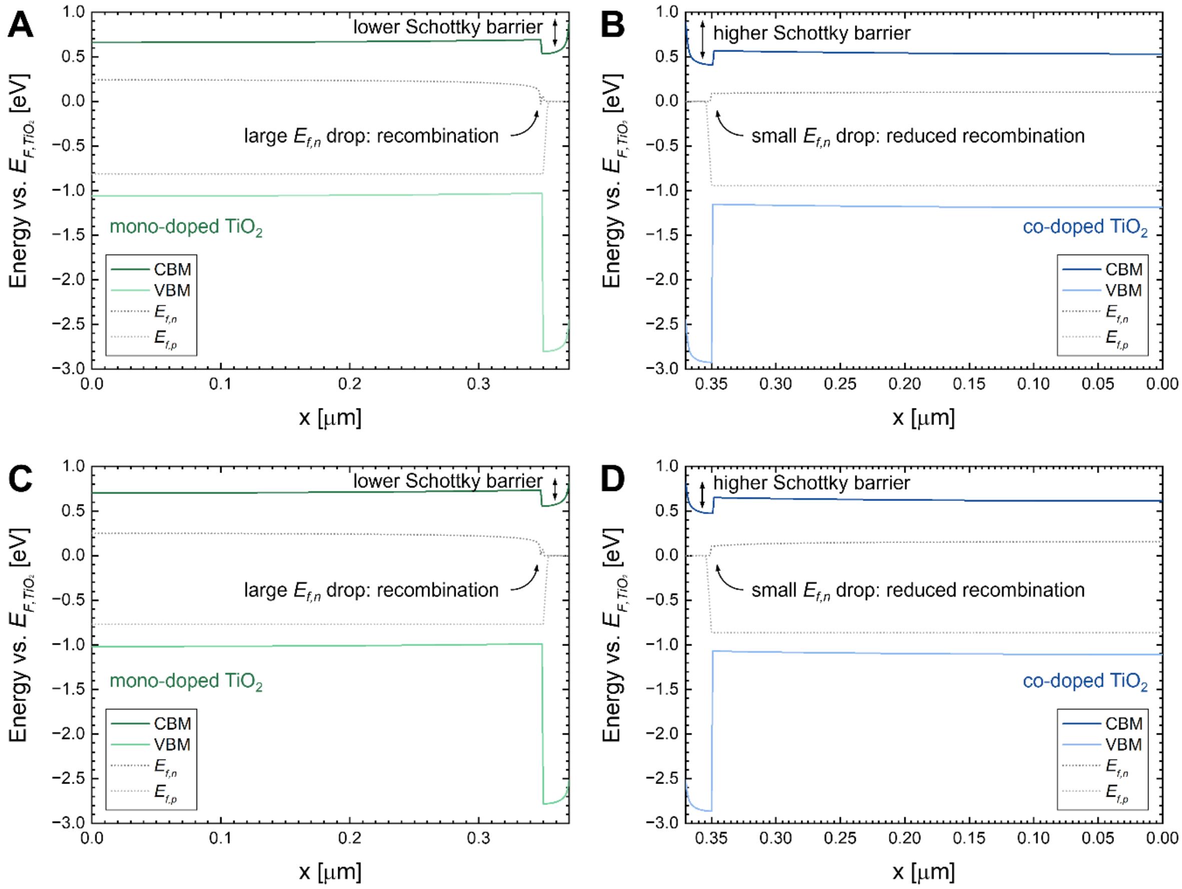

Fig. S24. Out-of-equilibrium band diagrams obtained with the parameters listed in **Tab. S5** to **Tab. S8**. The height of the Schottky barrier increases from left to right due to larger $E_{tD} - E_{VBM}$ in TiO_2. **(A, B)** Band diagram without consideration of shallow traps in $CsPbI_3$ in junction with (A) mono-doped and (B) co-doped TiO_2. **(C, D)** Fits with consideration of shallow traps in $CsPbI_3$ in junction with (C) mono-doped and (D) co-doped TiO_2.

S4 References

[1] W. N. Hansen, G. J. Hansen; Standard reference surfaces for work function measurements in air, *Surface Science* 2001, 481, 172.

[2] S. P. Harvey, J. Messinger, K. Zhu, J. M. Luther, J. J. Berry; Investigating the Effects of Chemical Gradients on Performance and Reliability within Perovskite Solar Cells with TOF-SIMS, *Advanced Energy Materials* 2020, 10, 1903674.

[3] a) F. Schaefers, M. Mertin, M. Gorgoi; KMC-1: A high resolution and high flux soft x-ray beamline at BESSY, *Review of Scientific Instruments* 2007, 78; b) M. Gorgoi, S. Svensson, F. Schäfers, G. Öhrwall, M. Mertin, P. Bressler, O. Karis, H. Siegbahn, A. Sandell, H. Rensmo, W. Doherty, C. Jung, W. Braun, W. Eberhardt; The high kinetic energy photoelectron spectroscopy facility at BESSY progress and first results, *Nuclear Instruments and Methods in Physics Research Section A: Accelerators, Spectrometers, Detectors and Associated Equipment* 2009, 601, 48.

[4] R. Félix, M. Gorgoi, R. G. Wilks, M. Bär; Hard x-ray photoelectron spectroscopy at a soft x-ray source: Present and future perspectives of hard x-ray photoelectron spectroscopy at BESSY II, *Journal of Vacuum Science & Technology A* 2021, 39.

[5] a) S. Tanuma, C. J. Powell, D. R. Penn; Calculations of electron inelastic mean free paths. V. Data for 14 organic compounds over the 50–2000 eV range, *Surface and Interface Analysis* 1994, 21, 165; b) S. Tougaard, Quases-Tougaard Inc., Odense, Denmark 2002.

[6] M. Wojdyr; Fityk: A General-Purpose Peak Fitting Program, *Journal of Applied Crystallography* 2010, 43, 1126.

[7] R. Nyholm, N. Martensson, A. Lebugle, U. Axelsson; Auger and Coster-Kronig broadening effects in the 2p and 3p photoelectron spectra from the metals 22Ti-30Zn, *Journal of Physics F: Metal Physics* 1981, 11, 1727.

[8] M. B. Trzhaskovskaya, V. I. Nefedov, V. G. Yarzhemsky; Photoelectron Angular Distribution Parameters for Elements Z=1 to Z=54 in the Photoelectron Energy Range 100–5000 eV, *Atomic Data and Nuclear Data Tables* 2001, 77, 97.

[9] M. P. Seah, *Journal of Electron Spectroscopy and Related Phenomena* 1995, 71, 191.

[10] T. Unold, L. Gütay, Photoluminescence Analysis of Thin-Film Solar Cells, in *Advanced Characterization Techniques for Thin Film Solar Cells*, Vol. 1, Eds: D. Abou-Ras, T. Kirchartz, U. Rau, 2016, pp. 275.

[11] H. Köbler, S. Neubert, M. Jankovec, B. Glažar, M. Haase, C. Hilbert, M. Topič, B. Rech, A. Abate; High-Throughput Aging System for Parallel Maximum Power Point Tracking of Perovskite Solar Cells, *Energy Technology* 2022, 10, 2200234.

[12] a) J. F. Moulder, J. Chastain, *Handbook of X-ray Photoelectron Spectroscopy: A Reference Book of Standard Spectra for Identification and Interpretation of XPS Data*, Physical Electronics Division, Perkin-Elmer Corporation, 1992; b) I. C. Tran, R. Félix, M. Bär, L. Weinhardt, Y. Zhang, C. Heske; Oxidation of Titanium-Decorated Single-Walled Carbon Nanotubes and Subsequent

Reduction by Lithium, *Journal of the American Chemical Society* 2010, 132, 5789.

[13] Y. Yuan, G. Yan, C. Dreessen, T. Rudolph, M. Hülsbeck, B. Klingebiel, J. Ye, U. Rau, T. Kirchartz; Shallow defects and variable photoluminescence decay times up to 280 µs in triple-cation perovskites, *Nature Materials* 2024, 23, 391.

[14] T. Chen, J. Xie, P. Gao; Ultraviolet Photocatalytic Degradation of Perovskite Solar Cells: Progress, Challenges, and Strategies, *Advanced Energy and Sustainability Research* 2022, 3, 2100218.

[15] S. X. Tao, X. Cao, P. A. Bobbert; Accurate and efficient band gap predictions of metal halide perovskites using the DFT-1/2 method: GW accuracy with DFT expense, *Scientific Reports* 2017, 7, 14386.

[16] K. J. Prince, M. Nardone, S. P. Dunfield, G. Teeter, M. Mirzokarimov, E. L. Warren, D. T. Moore, J. J. Berry, C. A. Wolden, L. M. Wheeler; Complementary interface formation toward high-efficiency all-back-contact perovskite solar cells, *Cell Reports Physical Science* 2021, 2, 100363.
